# Human Enamel Formation: A Scoping Review for Oral Health Professionals

**DOI:** 10.3390/dj14070421

**Published:** 2026-07-09

**Authors:** Patrick Unterbrink, Bernhard Ganss, Hardy Limeback, Birte Hollmann, Pascal Fandrich, Ingo Winschel, Erik Schulze zur Wiesche, Bennett Tochukwu Amaechi, Malgorzata Pawinska, Elzbieta Paszynska, Joachim Enax

**Affiliations:** 1 Research Department, Dr. August Wolff GmbH & Co. KG, 33611 Bielefeld, Germany; 2 Faculty of Dentistry, University of Toronto, Toronto, ON M5G 1G6, Canada; 3 Research Department, Dr. Kurt Wolff GmbH & Co. KG, 33611 Bielefeld, Germanyjoachim.enax@drwolffgroup.com (J.E.); 4 Department of Comprehensive Dentistry, University of Texas Health San Antonio, 7703 Floyd Curl Drive, San Antonio, TX 78229, USA; amaechi@uthscsa.edu; 5 Department of Integrated Dentistry, Medical University of Bialystok, 15-276 Bialystok, Poland; 6 Department of Integrated Dentistry and Endodontics, Poznan University of Medical Sciences, 60-812 Poznan, Poland; paszynska@ump.edu.pl

**Keywords:** enamel, amelogenesis, biomineralization, enamel matrix proteins, MMP20, KLK4, hydroxyapatite, scoping review, biomimetic, ameloblast, enamel defects, remineralization

## Abstract

**Background**: Tooth enamel is the hardest and most highly mineralized tissue in the human body. It serves as a protective barrier against chemical, mechanical, and microbial challenges. Despite its durability, enamel remains vulnerable to developmental and posteruptive defects such as fluorosis, hypomineralization, and amelogenesis imperfecta (AI). For oral health professionals, a clear understanding of the biological and molecular mechanisms underlying enamel formation is essential for advancing preventive and therapeutic strategies in clinical practice. This review synthesizes current knowledge on enamel formation, with emphasis on its cellular, molecular, and structural determinants, and discusses clinically relevant disruptions as well as emerging biomimetic approaches. **Methods**: This scoping review was conducted according to the PRISMA-ScR guidelines. A systematic literature search of the mechanisms of enamel formation was performed via Embase and Medline. Titles and abstracts were screened independently by three authors. Studies that primarily addressed enamel defects were excluded from the systematic synthesis; however, these studies were retained for narrative discussion. Following the screening process, 92 publications met the inclusion criteria and were incorporated into the thematic synthesis. **Results**: Enamel formation is a complex, multistage process involving epithelial–mesenchymal interactions and the sequential activity of ameloblasts during presecretory, secretory, transition, and maturation stages. Key mechanisms include the secretion of enamel matrix proteins (e.g., amelogenin, ameloblastin, and enamelin), proteolytic processing by enzymes such as MMP20 and KLK4, and controlled ion transport, leading to hydroxyapatite crystal growth and organization into rod and interrod structures. The structural arrangement endows enamel with exceptional mechanical resistance. Narrative sections address “What can go wrong?”, summarizing genetic, epigenetic, and environmental causes of fluorosis, hypomineralization, and amelogenesis imperfecta, and other developmental defects, whereas “What can we learn from nature?” highlights biomimetic strategies. **Conclusions**: Human enamel formation is a highly coordinated biomineralization process regulated at the cellular, structural, and molecular levels. Disruptions in these processes underlie major enamel pathologies. Integrating mechanistic insights from natural enamel development with emerging biomimetic technologies offers promising avenues for prevention, diagnosis, and treatment in dentistry. This review provides oral health professionals with a biologically grounded framework to guide evidence-based management of enamel-related conditions.

## 1. Introduction

### 1.1. Importance of Enamel in Biology and Dentistry

**Figure 1:**
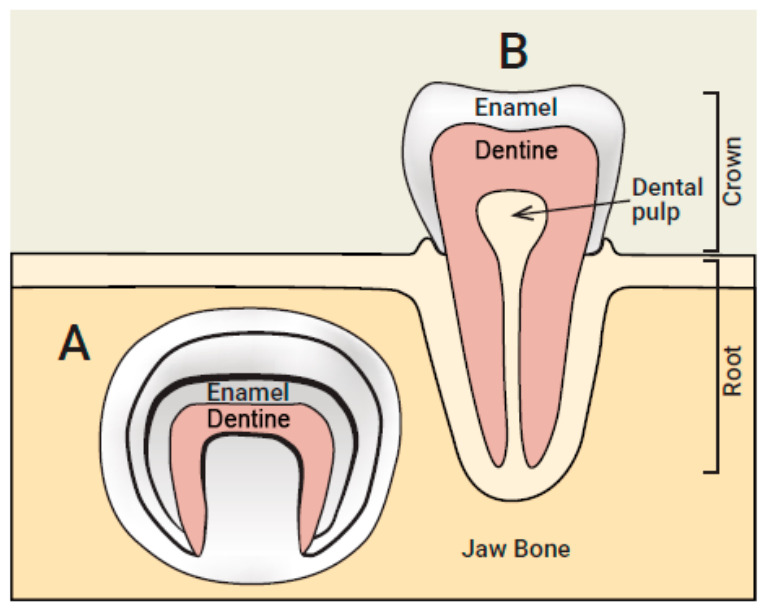
Schematic representation of a tooth within the jawbone with two different appearances. (**A**) Schematic of the process of tooth development before eruption, with the key tissues enamel and dentin depicted. (**B**) Schematic illustration of a tooth after eruption, with a root, crown and dental pulp. Adapted from Hubbard 2002 [2].

A comprehensive understanding of human tooth enamel, its formation, structural organization, and susceptibility to caries and erosion is highly important for dental practitioners aiming to deliver evidence-based preventive oral healthcare. As the hardest substance in the human body, enamel protects dentin and dental pulp from mechanical stresses (e.g., masticatory forces), chemical assaults (e.g., acids from food or bacterial metabolism), temperature fluctuations, and bacterial penetration (Figure 1A,B). If properly formed and cared for, it can withstand these challenges over a lifetime [1]. However, this first line of defense for teeth is sensitive to acid challenges and undergoes attrition with age. Dietary habits, including acidic food and beverages and sugar-rich nutrients, can further compromise the integrity of enamel.

These challenges are further underscored by the global burden of enamel caries. According to the World Health Organization (WHO), approximately two billion people still suffer worldwide from dental caries in their permanent dentition despite increased education, public health intervention, and the availability of oral care products. These findings emphasize the urgent need for preventive and regenerative strategies in dentistry [3]. In addition to caries and erosion, which occur after tooth eruption, the prevalence of certain developmental enamel defects, such as hypomineralization (chalky teeth, previously denoted as MIH [molar incisor hypomineralization] and, more recently, MH [molar hypomineralization]) and dental fluorosis, is increasing within the global population. The global prevalence of chalky teeth is approximately 13.5% [4], and the incidence of fluorosis has been reported to be as high as 65%, namely, in a cohort of 12–15-year-olds in the United States [5]. These enamel malformations not only lead to aesthetic problems but also pose significant clinical challenges due to their impact on enamel integrity, as they increase susceptibility to caries, dentin hypersensitivity, and posteruptive enamel breakdown.

In addition to enamel being the hardest tissue in the human body, Bartlett described its hardness as being between that of iron and carbon steel. It also stands as a remarkable example of biological mineralization (biomineralization) [6,7,8]. Mature human enamel consists almost entirely (approximately 97%) of calcium phosphate minerals in the form of hydroxyapatite (HAP) crystals, a low amount of organic material (approximately 1.5%), and water (approximately 1.5%) [9,10,11,12,13,14,15,16,17].

#### Amelogenesis

The extraordinarily high degree of mineralization of enamel, which is formed under physiological conditions, results from a fascinating, coordinated and highly regulated biomineralization process known as amelogenesis [18,19,20,21]. Duverger and Lee described the process of enamel formation aptly as ‘an exceptionally intricate, (…) multistep differentiation process that is orchestrated by a highly complex genetic program regulating both intracellular and extracellular interactions throughout amelogenesis’ [17]. Amelogenesis is governed by specialized epithelial cells (ameloblasts), which secrete a protein-rich enamel matrix and guide the whole process of enamel crystallite formation and maturation through different stages [17,22]. The two main phases of amelogenesis are secretion and maturation, which can be further divided into substages for explanation [14].

Once erupted, the enamel cannot undergo cellular repair, in contrast to dentin and bone, but it can be remineralized through the precipitation of calcium phosphate from saliva as well as by remineralizing agents from oral care products [23]. The hierarchically organized prismatic structure of the HAP crystals in the enamel, consisting of so-called rods and interrods, is characteristic of many mammals, including humans. The three-dimensional packing and decussation of these prism structures are essential for the outstanding mechanical properties of enamel [24].

Owing to the high degree of mineralization, the enamel represents the first line of defense of the tooth against acids, abrasive forces, and microbial invasions and thus protects the dentin. This barrier, if cared for properly, is biologically engineered to remain functional throughout life [17,25]. This is the reason why preventive dentistry has such significance in the maintenance of oral health.

### 1.2. Review Motivation

This scoping review synthesizes the contemporary understanding of the mechanisms underlying tooth enamel formation by integrating advances across multiple disciplines. It comprises two complementary components: first, a systematic literature review conducted in accordance with PRISMA-ScR guidelines [26] to delineate the biological processes driving enamel development; second, a narrative discussion that contextualizes selected clinically relevant enamel malformations. By concisely presenting current mechanistic insights, this review is expressly tailored to informing dentists and other oral health professionals in clinical practice.

Several literature reviews on enamel formation have already been published [27,28,29,30]. Additionally, Duverger and Lee provided valuable insights into genetic variations affecting amelogenesis and the resulting impairments [17]. The present review aims to provide a more practical and integrated understanding of the key steps in amelogenesis, combining biological detail with clinical relevance. Specifically, the objectives of this study are to elucidate important biological pathways involved in enamel formation, as well as the formational stages in ameloblasts, detail the proteins involved, and explore the epigenetic factors influencing amelogenesis. By achieving this goal, specific variations and defects in enamel formation can be discussed in a relevant context.

In the first section, this review presents and discusses the results of a systematic literature search on the cellular mechanisms of enamel formation, including the embryological stages of epithelial development and the critical pathways governing these processes. The stages of amelogenesis are subsequently discussed in detail by describing the lifecycle of ameloblasts, protein matrix secretion, structural components of enamel, growth of hydroxyapatite crystals, and rod/interrod structure characteristics of mature enamel. Following this detailed description, the molecular and genetic mechanisms of these stages are discussed, emphasizing the roles of key proteins and proteinases, the importance of cellular pH regulation, ion transport mechanisms, and genetic and epigenetic controls. Afterwards, potential disruptions during enamel formation are presented in a narrative way, providing insights into variations associated with AI, environmental influences, and clinically relevant enamel defects such as hypomineralization and fluorosis in Section 6.1. Finally, insights gained from natural enamel formation processes are discussed, particularly regarding enamel remineralization and material development, in Section 6.2.

Through this review, we provide the necessary background knowledge to help understand the biological and chemical reasons behind acquired or developmental enamel defects. We expect this knowledge to guide a confident and evidence-based decision-making process during the clinical management of enamel pathologies.

## 2. Materials and Methods

### 2.1. Approach and Search Strategy

The primary objective of this review is to elucidate the physiological and molecular mechanisms underlying enamel formation. Accordingly, the initial literature search strategy was designed to target this specific area by using the following search terms for abstract (ab), title (ti), and full text: ab(“enamel formation” OR “enamel development”) OR ti(“enamel formation” OR “enamel development”) OR amelogenesis. To maintain a focused scope for the systematic part, studies primarily addressing enamel defects were excluded. To achieve this goal, exclusion criteria specifically targeting such publications were defined as follows: ti (imperfecta OR fluorosis OR defect* OR cell OR hypoplasia OR MIH OR pathology OR syndrome OR caries OR dysplasia OR abnormal* OR hyperplasia OR malformation OR disease* OR disturbed OR impaired) OR ti (hypomineralization OR hypomineralization) OR ab (hypomineralization OR hypomineralization). Although such studies offer important insights, they often investigate pathological conditions resulting from genetic mutations or environmental influences, which fall outside the normal developmental framework. Nevertheless, in the second part, such a malformation will be discussed in a narrative way.

The Embase and Medline databases were accessed through Dialogue Proquest^®^ (2025 ProQuest LLC, Ann Arbor, MI, USA) on 17 February 2025 to identify eligible publications. The results of the initial search were then combined with the predefined exclusion criteria and further filtered to include only reviews, systematic reviews, and meta-analyses written in English.

No automation tools were employed during the subsequent selection process. Three authors (B.H., J.E. and P.U.) independently reviewed the resulting titles according to the predefined eligibility criteria and individually classified each title as “include,” “exclude,” or “uncertain”. After performing this independent review, the assessments were compared, and titles with discrepancies or unanimous uncertainty were subjected to a collective abstract screening by all three authors. This procedure led to the final set of publications selected for inclusion in this review. To facilitate the synthesis, two authors (J.E., P.U.) independently evaluated the selected studies to determine the topics covered by each publication. Following this independent evaluation, the authors’ assessments were compared to reach consensus on thematic groupings.

No data items are reported, no bias assessments are conducted, and no effect measures are presented. Instead, the literature synthesis follows the thematic approach outlined above.

### 2.2. Results of the Literature Search

Searching Medline yielded 3507 results, and Embase yielded 3289 results, resulting in a total of 6796 publications. Using the developed filter for defects, 6514 publications related to enamel defects or deficits during the formation process were excluded. Another 93 publications were excluded because they were duplicates.

**Figure 2:**
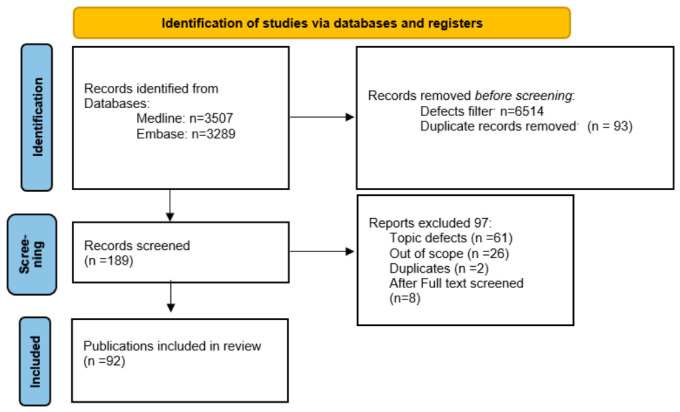
PRISMA flow diagram for the search. Identification, screening and inclusion process. Source: Page MJ, et al. BMJ 2021; 372: n71. doi: 10.1136/bmj.n71. This work is licensed under CC BY 4.0. To view a copy of this license, visit https://creativecommons.org/licences/by/4.0/ (accessed on 13 November 2025).

Accordingly, a total of 189 records were screened by three independent authors for eligibility. Of these, 61 were excluded because they fell within the scope of enamel defects or closely related defects, 17 were excluded because they were out of scope, and another 2 were duplicates that were not found during identification. After full-text screening, 8 more publications did not match the scope and were excluded. Finally, 92 publications were included as the basis for this review (Figure 2).

Each study reference is provided in the Supplementary Materials. As we are not synthesizing any results from different studies quantitatively, we do not report these items. The presented review was not registered in advance. The complete PRISMA-ScR checklist is available in the Supplementary Materials.

## 3. Cellular Basis of Enamel Formation—Embryological Stages

Development of human primary dentition starts at the sixth embryological week. The development of the secondary dentition starts at 20 weeks in utero and 10 months after birth, whereas the molars start at week 20 in utero (first molar) and 5 years of age (third molar). The end of tooth development can be assumed to be the breakthrough of wisdom teeth around the sixteenth year of life [31,32,33]. In the following section, the initial phases of the tissue interactions, arrangement and building of the tooth crown blueprint are described. These early morphogenic stages take place before the formation of enamel. Although the mechanisms are complex and often not covered in much detail, we believe that a brief description will help in understanding the developmental defects of enamel.

The foundational concept of inductive interactions between germ layers (ectoderm, mesoderm and endoderm) was first introduced by Spemann and Mangold [31,34]. Thirty years ago, how the initiation and complexities of morphogenesis work was still a central problem in developmental biology [35]. Subsequent experimental studies revealed that such interactions are mediated by specialized signalling molecules, including wingless-Int proteins (Wnts), bone morphogenetic proteins (BMPs), fibroblast growth factors (FGFs), sonic hedgehog protein (SHH) and ectodysplasin A receptor (EDAR). These factors orchestrate essential developmental processes by modulating gene expression and the activity of transcription factors within specific target cells [36,37,38,39].

Odontogenesis, the process of tooth formation, also relies on reciprocal signalling events between epithelial and mesenchymal components [17,30,40,41]. Seminal tissue recombination experiments in the 1960s confirmed the pivotal role of the mesenchyme in determining tooth identity—whether a molar, canine or incisor—and demonstrated its ability to reprogram epithelial cells into odontogenic tissue. These dynamic bidirectional interactions not only influence the morphological characteristics of the developing tooth but also drive its differentiation into two critical cell types: mesenchyme-derived odontoblasts, which are responsible for dentin formation, and epithelium-derived ameloblasts, which synthesize the enamel matrix [33,38,42].

**Figure 3:**
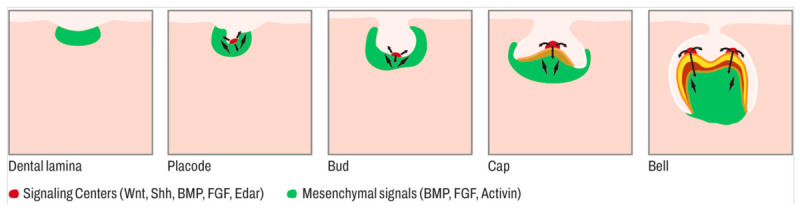
Embryological stages of tooth development. The developmental stages from the lamina, placode, bud, cap and finally bell stages are shown. First, the ectoderm (light cream) is thickened into a U-shaped epithelial band, forming the dental lamina above the nonneural crest-derived mesenchyme (dark cream). The neural crest-derived mesenchyme is shown in green, signalling centers are shown in red, and black arrows depict the signalling pathways. The ameloblast tissue is represented in orange, and the odontoblast tissue is represented in light brown. At the bell stage, already formed enamel is shown in yellow, and the dentin is shown in dark brown. Adapted from Lacruz et al. [27].

The ensuing stages of odontogenesis unfold through five embryologically distinct phases: the dental lamina, placode, bud, cap, and bell phases [11,30,32,35,37,41,43] (Figure 3). The actual development of the primary dentition starts by the sixth embryonic week, when bilateral regions of thickened ectoderm in the maxillary and mandibular arches give rise to U-shaped epithelial bands, termed the dental lamina (Figure 3), which are endowed with odontogenic potential [32,36,44]. These bands invaginate into the underlying mesenchymal primordia of the jaws, marking the onset of the placode stage. These placodes function as early signalling hubs, exhibiting strong expression of key morphogens such as SHH, BMP, FGF, and WNT, which collectively direct progressive epithelial ingrowth and initiate subsequent developmental transitions. Simultaneously, the odontogenic potential is transferred from the epithelial lamina to the adjacent neural crest-derived mesenchyme [36,38,40,45].

Around the eighth developmental week, ten discrete epithelial thickenings, referred to as tooth buds, emerge along the lateral margins of each dental lamina, constituting the bud stage [39]. These buds penetrate the surrounding mesenchyme and represent the primordia of the primary (deciduous) dentition, ushering in the cap stage [36]. Concomitantly, the primary enamel knot, a transient signalling center, is typified by high expression of signalling genes and exits the cell cycle via p21 induction. p21 induction refers to an increase in p21 protein levels, which act as a cell cycle regulator [46]. This orchestrates lateral epithelial proliferation, which is essential for cap formation [36]. During the transition from the cap to the bell stage, the enamel organ undergoes further morphogenetic folding, with the inner enamel epithelium elongating and defining the final contours of the future crown [47]. The secondary enamel knots emerge as transient signalling centers that pattern the positions of future cusps through spatially restricted expression of SHH, BMP, and FGF family members [48,49].

In the early bell stage, histodifferentiation becomes prominent. The cells of the inner enamel epithelium differentiate into preameloblasts, whereas the peripheral cells of the dental papilla differentiate into preodontoblasts. Concurrently, the stellate reticulum expands, creating a cushioning extracellular matrix that supports enamel organ morphogenesis [11,33]. Subsequent morphogenetic folding of the enamel organ defines the definitive shape of the tooth crown in the bell stage [14,36]. For further reference and visual resources, see digitalhistology.org [50].

**Figure 4:**
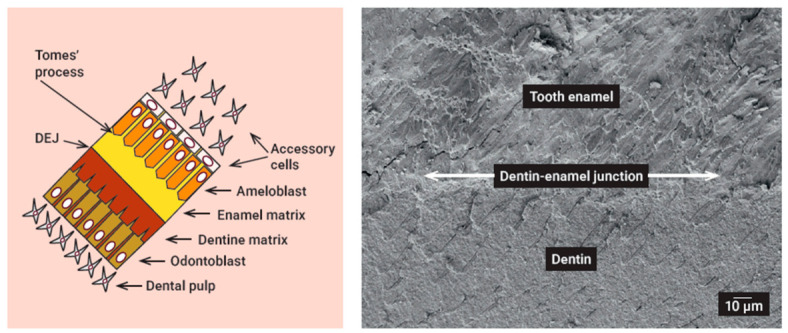
Overview of the dentin-enamel junction (DEJ). **Left**: schematic of the developing tissue layers, with odontoblasts and the dentin matrix as well as ameloblasts and the enamel matrix. The dental pulp and accessory cells are shown for illustration. Tome processes are shown as small tips at the ends of the ameloblasts. **Right**: SEM image (magnification: 1000×) of a developed human tooth, showing the DEJ. Top: enamel; bottom: dentin. Adapted from Hubbard 2002 [2,54].

The late bell stage is characterized by the polarization of preodontoblasts and preameloblasts, increased synthetic organelle development, and the initiation of the secretory phenotype. Vascularization of the dental papilla intensifies, ensuring an adequate nutrient supply for the forthcoming matrix secretion phase (Figure 3) [14,28]. During the late bell stage, the basement membrane still separates the differentiating odontoblasts of mesenchymal origin from the preameloblasts derived from the epithelium. This boundary later gives rise to the dentino-enamel junction (DEJ; Figure 4) [11]. Figure 4 shows scanning electron microscopy images of the DEJ. Scanning electron microscopy (SEM) is an electron beam-based characterization technique in which the backscattered electron fraction emitted from a sample is modulated by the crystal orientation and the material mean atomic number from the emission volume, with performance at finer scales. Field-emission SEMs (particularly cold-field emitters) provide the highest signal and large probe current densities that, when combined with efficient electron detectors, achieve high signal-to-noise ratio images with nanoscale spatial resolution [51]. Techniques such as these facilitate gaining more insight into the complex tooth structure [52,53].

As odontoblasts begin secreting a predentin matrix, the basement membrane is degraded, permitting direct contact between ameloblast precursors and predentin [11]. This contact is believed to act as a critical signal, triggering terminal differentiation of preameloblasts into enamel-producing ameloblasts [28,55]. In humans, this differentiation commences at cusp tips and signifies the initiation of enamel formation [14,56].

## 4. Results

### 4.1. Ameloblasts

Understanding ameloblast function is essential for oral health practitioners, as it provides critical insights into the etiology, prevention, and treatment of enamel defects that are commonly encountered in daily dental practice.

Ameloblasts and their morphological and functional changes during amelogenesis, which take place during the advanced/late bell stage, constitute the most important basis for understanding the process of mineralization by calcium and phosphate and thus the development of HAP-based enamel. These different stages and related changes in ameloblast formation have been described previously [7,57,58]. Furthermore, these stages and the underlying molecular mechanisms are also prone to defects visible in everyday oral care, such as enamel malformations, which will be the topic of Section 6.1.

Amelogenesis requires approximately four to five years to reach completion, with the maturation of enamel accounting for nearly two-thirds of this period [19]. While this process has been characterized as comprising three distinct stages, contemporary research more commonly distinguishes four sequential phases: presecretory, secretory, transition, and maturation [15,16,30,32]. This refined classification provides a useful framework for understanding the phenotypic transformations of ameloblasts, as they modulate their functional roles [15,28,41,55,56].

Throughout all stages of enamel formation, ameloblasts are central to the process. In the early phases, they undergo pronounced morphological and functional modifications to produce large quantities of secretory vesicles, facilitating enamel matrix deposition. In later stages, their role shifts toward the removal of enamel proteins and the promotion of crystal growth and mineralization during enamel maturation [59].

### 4.2. Phases of Amelogenesis

In the following, the single stages of amelogenesis are described, considering mainly the changes in ameloblast form and function. The molecular mechanisms and important polypeptides involved in creating the enamel are described in Section 5.2.

#### 4.2.1. Presecretory

**Figure 5:**
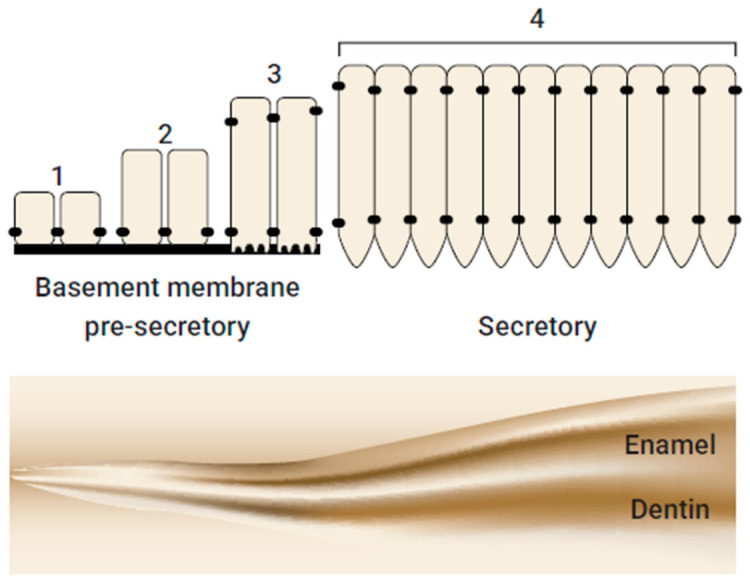
Overview of the morphological changes in ameloblasts during the depicted stages of amelogenesis (top), and the tissue changes are schematically drawn below. The presecretory stage involves three subchanges of the ameloblast: (1) Pre-ameloblasts elongate (2) and start to solve the basement membrane (3). A second distal cell connection is formed (3 black dots). Secretory (4) stages involve elongated ameloblasts with fully developed Tomes’ process at the bottom. The tissue composition changes during these stages, while enamel matrix proteins are secreted. Both enamel and dentin are produced at the same time.

**Figure 6:**
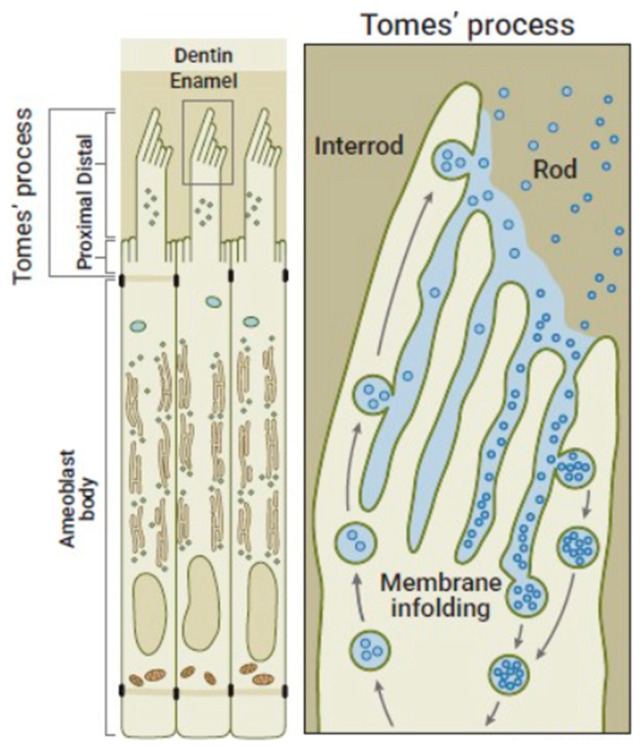
Secretory activity during the secretory stage. During the secretory stage, ameloblasts develop a Tomes’ process at their apical membrane, which comprises a proximal and a distal segment. The proximal portion is involved in the formation of interrod enamel, whereas the distal portion contributes to rod enamel formation. Vesicle fusion events are observed at the apical membrane near the sites of rod-shaped enamel elongation. Numerous vesicles either fuse with (secretion) or bud from (endocytosis) membrane infoldings located at both the proximal and distal regions of Tomes’ process. Adapted from Pham 2017 [59].

During the initial phase of amelogenesis, preameloblastic cells differentiate into ameloblasts. As mentioned earlier, this differentiation occurs upon contact with predentin [15,17,60]. At this point, enamel proteins are already secreted above the predentin [11,32], resulting in irregularities in the dentin. During this phase, the whole cell is transformed for secretion: the cell bodies begin to elongate (Figure 5), and the organelles take their functional niches within the cell arrangement; e.g., the nucleus and mitochondria polarize to the proximal end [11,30,61]. During this stage, the volume of the Golgi complex and the amount of rough endoplasmic reticulum (rER) surrounding it increase, as these proteins are synthesized for the enamel matrix [61]. A second cell connection via tight junctions (Figure 5; 3) is formed at the distal end, which finally polarizes the ameloblast into a cell body and a distal structure where the majority of secreted proteins are released, the so-called “tome process” (Figure 6) [9,11,13,15,28,55,56,62,63]. Importantly, two different parts of the Tomes process produce two slightly different enamel structures (rod/interrod), which will be described later.

#### 4.2.2. Secretory

This stage is characterized by the secretion of a vast amount of enamel matrix proteins and minerals through vesicles (Figure 6) [29,63]. To secrete this large quantity of proteins, the Golgi complex and the rER were enlarged in the previous stage. The RNA is translated at the rER and packed into secretory vesicles, which are further transported to the Tomes process and released against the dentin [64]. During this stage, ameloblasts secrete many proteins, such as amelogenin, ameloblastin, enamelin and metalloproteinases, whose functions are described later. However, the two key roles are to provide a blueprint for the formation of enamel crystallites and prevent them from fusing into a solid crystal structure [7,22,29,32,56,65].

The ameloblasts subsequently migrate away from the DEJ and are separated from this interface by the enamel matrix they produce. Owing to the particular spatial arrangement of Tomes, further formed enamel proteins are secreted from two different sites (s. Figure 6). The distal site forms the rod structure, and the proximal site forms the interrod structure. Both structures differ only in terms of the resulting calcium phosphate crystal orientation [9,33,61,64,66]. A nonsecretory part of the cell membrane gives rise to a space between the rod and interrod structures [11].

**Figure 7:**
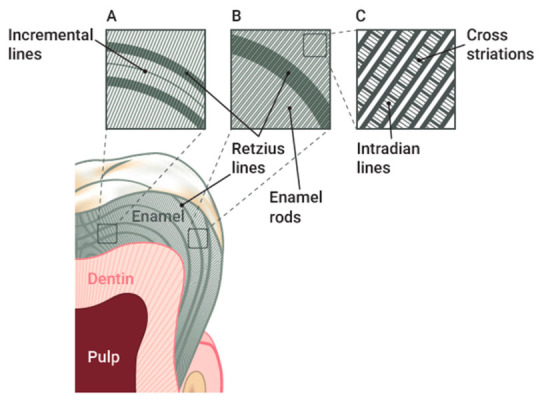
Micropatterns of mature tooth enamel and their origin. Different zoom perspectives on the whole enamel, incremental lines, Retzius lines and enamel rods as well as cross striations and intradian lines. Adapted from (Wu, Li et al. and Schneider et al. 2018) [67].

The biomineralization of enamel is not a continuous process but is based on circadian rhythm, resulting in the gradual growth of apatite, which is visible through cross stripes in the crystals (Figure 7). Another more prominent example of this incremental growth activity is known as the ‘striae of Retzius’, indicating a change in secretory activity approximately every ninth day (Figure 7). The most prominent example of these environmental influences on ameloblast activity is the ‘neonatal line’ that forms at birth, when ameloblast activity is temporarily altered due to stress during birth and separation from the mother [11,13,67].

At the end of the secretory phase, the enamel consists of approximately 5% water, 29% minerals and 66% protein and is soft, often described as ‘cheese-like’ and consistent [7,33].

#### 4.2.3. Transition

**Figure 8:**
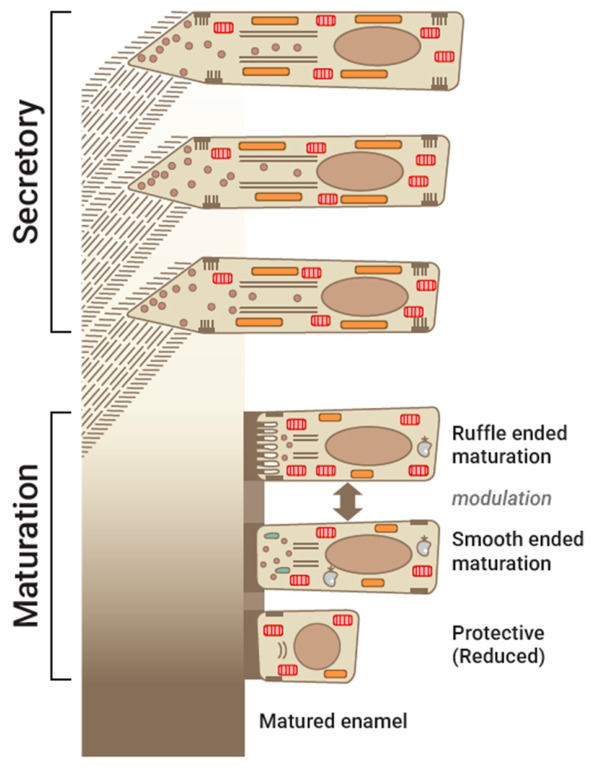
Overview of the changes during the two main processes: secretion and maturation. In particular, functional changes in the organelles within the ameloblast cell body are depicted here. The color gradient from top to bottom indicates the degree of ripening of the enamel until it matures.

The transition stage is characterized by morphological changes in ameloblasts between the secretory and maturation stages, as these two stages have different primary functions (Figure 8). At this point, the ameloblasts stop moving and retract the Tomes process [7,19]. Furthermore, they shrink in length and become wider, reorganizing their cell organelles (Figure 8; Maturation) [11], resulting in reduced secretory activity [28]. They begin to modulate their distal (enamel matrix-facing) membrane between “ruffled” and “smooth-ended” appearances to accomplish their needs during maturation. Approximately 25% of ameloblasts die during this stage through apoptosis [27,58].

#### 4.2.4. Maturation

After the transition phase, the enamel is far from complete in terms of composition and hardness. Therefore, this last stage in the process of enamel formation, before tooth eruption, is necessary to mature the crystals to harden the enamel. This maturation process takes place through increases in the width and thickness of the apatite crystals, which substitute for the removed organic matrix. During this maturation stage, kallikrein-related peptidase 4 (KLK4) is released to cleave residual matrix proteins and peptides [16,32,56,68].

Duverger and Lee provide an extensive summary of what they call ‘(…) the mission of the ameloblasts during maturation: (1) getting rid of enamel matrix proteins, (2) transfer all the minerals necessary for maturation and (3) create the perfect environment for crystallization’ [17]. The maturation process itself can take up to two-thirds of the whole time needed for enamel formation. During this stage, calcium, phosphate, and bicarbonate ions are transported to the crystals, and water and proteins (EMPs) are removed [69]. The exact mechanisms and influences of ion transport have subsequently been described [11,32].

**Figure 9:**
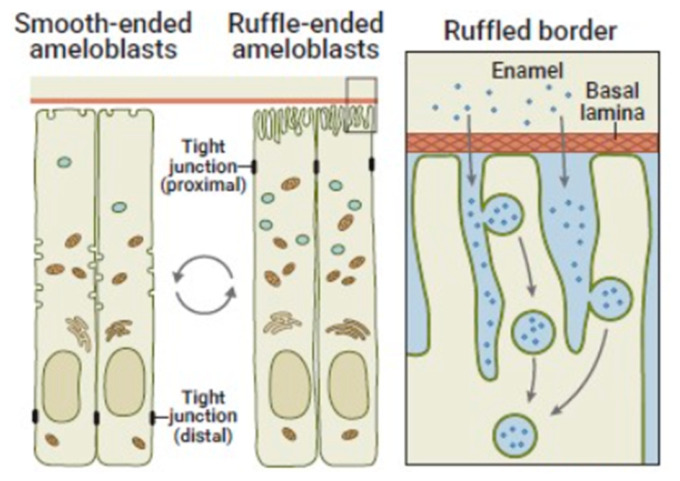
Endocytic activity during the maturation stage. In the maturation stage, degraded enamel matrix proteins are internalized by ameloblasts. These cells cyclically modulate between smooth-ended and ruffle-ended apical membranes. Approximately 80% of ameloblasts in this stage exhibit ruffle-ended borders characterized by pronounced membrane invaginations. Degraded enamel proteins diffuse through the spaces between the convoluted enamel tubules and are resorbed via vesicle-mediated endocytosis. Adapted from Pham, 2017 [59].

Maturation is characterized mainly by the alternating ruffle- and smooth-ended phenotypes of ameloblasts (RA, SA) (Figure 9) [16,55,59,63]. After approximately two hours in smooth-ended form, they turn ruffle-ended for 8.5 h [70]. This phenotypic change is important for regulating the pH within the area where enamel undergoes mineralization and establishing a Ca^2^+ gradient from the bloodstream to ameloblasts [32]. During SA, water and the organic matrix are removed, and during RA, ion transport and pH regulation take place.

Dynamic changes in junctions play important roles, as they are tight at the apical site during RA and leaky both apically and distally during SA [63]. In addition, they ensure the endocytosis of the debris of the EMPs [16]. Furthermore, a variety of proteinases, as well as endocytosis by ameloblasts, are involved in the degradation of EMPs, ultimately resulting in a mineral content of 96%, a water content of ~3% and an organic content of ~1% [17,59]. Finally, approximately 50% of ameloblasts undergo apoptosis at this stage [55].

Before investigating proteins involved in amelogenesis, it is necessary to analyse the structural properties of enamel itself and how crystallization works in general. Afterwards, the most prominent proteins involved in the enamel matrix are introduced, and their functionalities are discussed.

## 5. Structural Properties of HAP and Molecular Mechanisms in Enamel Formation

As depicted above, amelogenesis is a complex process forming enamel, with different time frames for different stages as well as different mechanisms and organelles involved. The following focuses first on the resulting structure of enamel and second on the molecular mechanisms, especially the proteins and genes involved, during this process, as well as the mineral transport mechanisms.

### 5.1. Structural Organization of Enamel

While the initial enamel deposited on dentin consists of aprismatic minerals that mature under the guidance of Tomes’ processes, the resulting enamel exhibits varying appearance and characteristics [71]. The aprismatic apatite is readily soluble in mild surroundings, whereas the prismatic apatite is much more robust. This becomes apparent as a sudden increase in carious lesions, reaching the dentin through the enamel, is observed [40,71].

**Figure 10:**
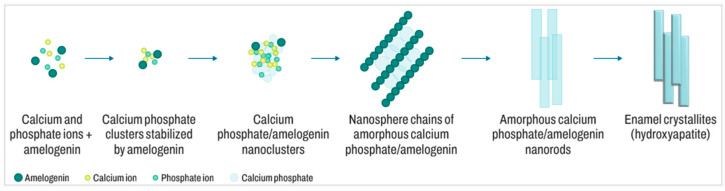
Schematic representation of enamel formation in sequential stages. Initially, calcium and phosphate ions interact with amelogenin, forming stabilized calcium phosphate clusters. These clusters further aggregate into calcium phosphate/amelogenin nanoclusters, which assemble into chains of amorphous calcium phosphate/amelogenin nanospheres. Continued assembly leads to the formation of amorphous calcium phosphate/amelogenin nanorods, which finally crystallize into enamel crystallites composed of hydroxyapatite (adapted from Lacruz et al. 2017 [27]).

The mechanism of enamel crystallite formation is composed of sequential stages (Figure 10). This process basically takes place at the mineralisation front [72]. The necessary ions for the formation of HA, which are calcium and phosphate, are present and lead to the formation of amorphous calcium phosphate (ACP). In general, ACP serves as a precursor for the formation of hydroxyapatite during calcium phosphate crystallization. During amelogenesis, these ACP nanoclusters are stabilized by the protein amelogenin [73]. Subsequently, chains of nanospheres composed of amorphous calcium phosphate are formed, followed by the development of nanorods. In a further crystallization maturation process, the final enamel crystallites are formed. Taken together, this complex crystallization process leads to hierarchically organized nanostructures and microstructures in enamel. The organic matrix is almost completely degraded after enamel formation, resulting in a hydroxyapatite content of approximately 97% in mature enamel [74,75].

#### Rod/Interrod Structure

**Figure 11:**
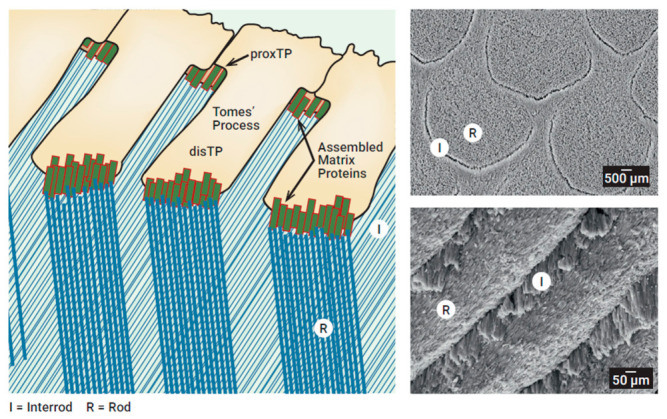
Overview of the rod/interrod structure and appearance analysed via SEM. **Left**: Schematic representation depicting the Tomes process in several ameloblasts. The distinction between proximal (proxTP) and distal (diTP) Tomes’ processes is shown. The green boxes indicate the assembled enamel matrix, which produces either a rod (brown) or an interrod (blue) enamel structure. Adapted from Habelitz, S. et al. 2015. **Top and bottom right**: SEM image of a human tooth at 20,000× magnification [54,71].

As described above, the secretion process results in two different HAP structures: rods and interrods (Figure 11). These different structures are thought to be responsible for the high resilience to external forces. They are thought to have slightly different orientations according to the type of secretion process (either proximal or distal end of the Tomes’ process), resulting in the so-called key-hole structure, but are aligned within each other, which can be analysed via SEM (Figure 11) [76]. Beniash et al. demonstrated that the latter does not hold true considering the orientation within each: the rods have a ‘sweet-spot’ of misorientation of approximately 30° of the single HAP crystals, which is the reason for the strong deflecting-crack properties of the enamel [77].

Interrods, in contrast, have a different structure. They seem to be co-oriented over millimeters, starting at the DEJ and continuing to the tooth cusps. With respect to the exact process leading to the misoriented rod crystals not yet known, Beniash et al. discussed three different phases potentially the cause: amorphous calcium phosphate precursors, ‘ion-by-ion’ precipitation of the crystals or the formation of nanoribbons as templates for the assembly of the crystals, as described by Habelitz et al. [75]. Nevertheless, the organic matrix seems to control crystal growth in such a tremendous manner that thermodynamics seems to be overcome [78].

### 5.2. Molecular Control of Amelogenesis

At the protein level, secretion of the enamel matrix is essential for the initial mineralization of enamel. The protein-, mineral-, and water-rich environment supports continuous enamel formation. More than 40 different proteins and proteinases are known to participate in distinct tasks during this process [2]. In this context, attention is focused on a subset of proteins with well-established roles: amelogenin, ameloblastin, enamelin, and tuftelin, as well as the proteases metalloproteinase 20 (MMP20) and KLK4 [15,63,64,79,80].

Genetic factors are equally important in the regulation of amelogenesis. Specific genes contribute both to the construction of intact enamel and to the differentiation of ameloblasts. Disruptions in these molecular mechanisms can result in various enamel defects that may increase susceptibility to caries [81]. A deeper understanding of these processes is therefore highly valuable for the development of preventive and therapeutic strategies in dental care.

Amelogenesis represents a typical biomineralization process in which inorganic components are organized into a highly ordered microstructure under the guidance of specific organic molecules, primarily proteins. In the secretory stage, enamel growth occurs through the deposition of matrix proteins and initial mineral formation. The maturation stage involves the progressive replacement of organic material with minerals, resulting in hardening of the material. This section focuses on the molecular mechanisms underlying these two key stages.

#### 5.2.1. Molecular Regulation in the Secretory Stage (Proteins)

During the secretory stage, ameloblasts produce and secrete several specific proteins into the growing enamel space, initially at the enamel–dentin interface. Ameloblasts then progressively recede from the interface with dentin as they continue to produce an organic extracellular matrix until the final thickness of the enamel has been reached. Three main structural enamel proteins, namely, amelogenin (approx. 90% of the organic material in the enamel matrix), ameloblastin (5–9%) and enamelin (1–5%), are secreted at this stage.

##### Amelogenin

Amelogenins (AMELs) are the most abundant proteins secreted by ameloblasts and are present in the enamel matrix [82]. The proteins are encoded on the X and Y chromosomes in humans and transcribed from both loci. AMEL makes up approximately 90% of the organic matrix during the secretory stage of enamel formation. With a molecular weight of 21.6 kDa, the protein monomer aggregates into supramolecular structures that have been described as nanospheres [79,83,84] or nanoribbons [85]. This aggregation is mediated by domains within the secondary structure of the protein and is dependent on the local pH and ion concentration [86,87]. The supramolecular arrangement of AMEL serves as a template or scaffold for the initial formation of enamel minerals. AMEL controls not only mineral deposition but also the transition from calcium phosphate mineral precursor phases, such as ACP, into the final HAP and its organization into an intricate and highly ordered three-dimensional structure.

##### Ameloblastin

Ameloblastin (AMBN), with approximately 5–9% organic matrix, is the second most abundant EMP in human enamel formation. AMBN is a multifunctional protein that regulates cell attachment, mineralization, ameloblast differentiation and the microstructural organization of enamel into rod and interrod elements [88]. Various domains in the 48.3 kDa protein are associated with protein–protein and protein–cell membrane interactions. Additionally, their cleavage products are located between the rods, which gives them the name “sheath proteins” or “sheathlin” [16]. Therefore, this protein is considered to play two different roles: one at mineralization and the other at rods [15].

##### Enamelin

Enamelin (ENAM), accounting for approximately 1–5% of the organic enamel matrix, is a relatively low-abundance protein, but its molecular mass is the largest at 128.8 kDa. The nucleation and transformation of calcium phosphates during enamel formation appear to be regulated by interactions of ENAM with AMEL (Jinhui2018), particularly at the DEJ [89], but little is known about the specific functions of ENAM and its domains in enamel development and mineralization [90]. The fact that ENAM is essential for enamel formation has been proven by multiple reports that associate mutations in the ENAM gene with various types of AI [91,92,93,94].

##### Tuftelin

Tuftelin (TUFT1) is another minor constituent of the developing enamel matrix [84,95]. The 44.3 kDa protein has been implicated in the control of enamel mineralization [96], but it performs other biological functions in epidermal tissues [97].

##### Processing of the Enamel Matrix: Proteolysis and Phosphorylation

Soon after the discovery and characterization of EMPs, secreted proteins were found to be processed by a number of post-translational modifications and/or proteolytic cleavage [98]. This is accomplished mainly by the matrix metalloproteinase MMP-20, which cleaves AMEL, AMBN and ENAM at specific sites [99]. In the case of AMEL, cleavage by MMP-20 produces fragments that control the slow transition from precursor minerals such as ACP into HAP [100,101]. This transition has to progress at a limited rate so that the microstructure of enamel can form properly. The same is true for AMBN [102,103] and ENAM [104]; thus, the organic matrix composition at this stage becomes rather complex. During the exploration of MMP20, it was thought to be exclusively produced during the enamel formation process and was therefore named ‘enamelysin’ [64]. It shows high affinity and substrate specificity for amelogenin [15] but also cleaves AMBN and ENAM [16]. In addition to being cleaved, EMPs undergo post-translational modifications at critical positions. For example, phosphorylation of the serine residue at position 16 in AMEL appears to be critical for its biological role [105]. AMBN and ENAM also undergo phosphorylation [106,107,108], and mutations in critical phosphorylation sites lead to AI [109]. Some of the enzymes that are involved in EMP phosphorylation (e.g., the casein kinase FAM20C or the cytoskeletal regulator FAM83H in concert with casein kinases) have been identified [110,111].

### 5.3. Molecular Regulation in the Maturation Stage (Proteinases)

Decades ago, it was already discovered that enamel contains vastly different amounts of proteins, comparing the secretory and maturation stages [18]. At the end of the secretory stage, the enamel matrix only contains approximately 30% mineral, with the remainder being organic material and water. While it is not well understood which signals trigger the transition from the secretory to the maturation stage, ameloblasts undergo dramatic changes during this transition and essentially change their phenotype from protein-producing cells to transport cells. The supporting cell types (stratum intermedium and stellate reticulum in the secretory stage) fuse to form the papillary layer at the maturation stage. The main events during enamel maturation are (1) the removal of almost the entire organic material and (2) the influx of vast amounts of calcium and phosphate ions as building blocks for the (almost) complete mineralization of the enamel. In addition, the formation of HAP generates protons and thus acidification of the enamel space, which needs to be neutralized to allow HAP deposition. Although we currently have a good understanding of the molecular mechanisms of the secretory stage, the events at the maturation stage are highly complex and incompletely understood [19,68]. The removal of the residual organic matrix is facilitated by the proteolytic cleavage of EMP fragments by the rather promiscuous serine protease kallikrein-related peptidase KLK4. This glycosylated enzyme is secreted into the enamel space at the transition and maturation stages and cleaves peptides into small fragments and single amino acids. However, how these amino acids are transported out of the enamel space, whether this occurs via passive diffusion or active transport, and how their removal is accomplished throughout the entire enamel layer remain poorly understood. On the other hand, several ion transporters that mediate the influx of calcium and phosphate ions into the enamel space have been identified [112,113], and some progress has been made in understanding the mechanisms that regulate pH [63].

#### 5.3.1. Ion Transport, Mineralization and Endocytosis

While this review focuses on the main mechanisms of ion channels and their role in dental enamel formation, more than 20 ion channels and different subtypes have been described in the literature [28,114]. Nevertheless, some major channelopathies will be explained in Section 6.1.

In addition to the transport of proteins through vesicles to the mineralization front, ion transport mechanisms also play crucial roles. These processes can be divided into two parts: one is the transport of ions to the mineralization front, already shown by Frank in 1979 [115], to be built into the enamel, and the other mechanism is the steady ion flux needed for active transport mechanisms. Ca^2+^ transport is needed in a steady fashion, as it is one of the main components of HAP [6,113]. The ameloblasts use channels, pumps and transporters to increase the amount of calcium needed [32]. This ion flux is necessary, as studies suggest, for example, a decrease in pH during maturation according to crystal nucleation. Thus, pH buffering is needed to keep the enamel intact [19,30,57,63,116].

#### 5.3.2. Role of Proteins in Deposition

Over the past two decades, additional proteins that are predominantly produced in relatively low amounts by ameloblasts at the maturation stage have been identified. These proteins belong to the family of secreted calcium-binding phosphoproteins (SCPPs) and include amelotin (AMTN), odontogenic-ameloblast associated protein (ODAM), odontogenesis-associated phosphoprotein (ODAPH), secreted calcium-binding phosphoprotein rich in proline and glutamine (SCPPPQ) 1 and follicular dendritic cell-secreted protein (FDC-SP). Although they are secreted into the enamel space, their localization is restricted to the interface between the mineralizing enamel matrix and the ameloblast cell. Several publications have confirmed that AMTN promotes HAP mineralization during the establishment of the densely mineralized enamel surface layer [117,118]. ODAPH may play a similar role [108], while recent work has indicated that ODAM and SCPPPQ1 may be involved in maintaining the attachment of cells to enamel minerals after tooth eruption [119,120,121]. The concept of a developmental continuum from ameloblasts to junctional epithelium cells that mediate dentogingival attachment has been known for some time, and proteins such as ODAM and SCPPPQ1 may be key to maintaining this attachment [25].

## 6. Developmental Defects and Outlook

### 6.1. What Can Go Wrong?

Any disruption in these steps, whether due to genetic mutations, environmental factors, systemic diseases, or nutritional deficiencies, can impair enamel quality or quantity. Such defects may manifest as hypomineralization, hypoplasia, or structural weaknesses, increasing susceptibility to dental caries, wear, and sensitivity. Understanding the causes and mechanisms of defective enamel formation is critical for early diagnosis, prevention, and the development of targeted therapeutic strategies [122]. Importantly, without studying the realm of “what can go wrong” in terms of aberrant protein function and genetic disturbances, many functions have not been discovered [123].

#### 6.1.1. Amelogenesis Imperfecta

AI is a group of enamel defects that occur due to genetic developmental defects. At each stage of the formation process [124], due to the critical function of certain proteins, these can be phenotypically characterized by a deficient enamel structure and appearance. These defects can affect deciduous as well as permanent teeth and manifest through yellowish colouration, increased sensitivity and an increased prevalence of caries. The cause of AI lies in mutations in specific genes responsible for the formation and mineralization of EMPs.

As comprehensively described, the process of enamel formation is a tightly regulated developmental process not only on the basis of the expression of certain proteins but also on the precise temporal and spatial regulation of genes encoding these proteins. Therefore, these genes, as well as epigenetic mechanisms, play crucial roles in amelogenesis, and perturbations at these levels can lead to changes in enamel quantity or quality, ranging from subtle hypomineralization to severe enamel malformations [27,81].

##### Genes and Associated Malformations

AMELX is the gene predominantly responsible for encoding amelogenin and is the only SCPP gene positioned on the X chromosome and the Y chromosome [16,28,125]. The expression of this protein stops after the maturation stage is finished [15,56]. Pathogenic variants cause X-linked forms of Ai [105]. Li et al. also showed, via gene-based analysis, that a certain gene variant of AMELX (rs17878486) is significantly associated with the risk of dental caries [126]. Additionally, mutations result in more surface texture and pronounced roughness [127]. ENAM is responsible for the expression of enamel, which is critical for the formation and elongation of HAP crystallites [91]. Thus, known mutations are associated with localized hypoplastic areas, normal hardness but quantitatively reduced enamel or a lack of surface texture, a smaller tooth size, and poor aesthetics [127]. AMBN encodes ameloblastin and is thus responsible for maintaining the rod/interrod boundary mutations that lead to hypoplastic AI. As such, it is equally important as amelogenin for enamel growth [15]. Chun et al. proposed a murine model in which overexpression leads to demarcated hypomineralized and opaque enamel, which is a fundamental feature of hypomineralization [128]. Another recent review revealed that enamel could also be completely missing [127]. ADAM10 encodes desintegrin and metalloproteinases (ADAMs), which are a family of membrane-bound zinc-dependent proteinases that cleave the extracellular domains of membrane-bound proteins. Mutations in this gene seem to cause discolored and softer enamel, with significantly reduced density and volume. According to the resulting rod pattern, it is assumed that the Ameloblasts showing this kind of genetic mutation do not move properly [90]. MMP20 mutations can result in hypomatured, ‘snow-capped’ enamel, since the enamel is not properly matured [127]. KLK4 mutations result in a diffuse pigmented, hypomatured enamel phenotype. Brown discolouration can be observed, and the enamel is soft [127]. Ambn plays a crucial role in maintaining the rod–interrod structure [16].

##### Epigenetic Influences

In general, epigenetic studies analyse the reversible DNA changes that regulate genetic transcription. As described above, epigenetic changes do not directly alter the sequence of nucleic acids. The main epigenetic processes include DNA methylation and histone post-translational modifications, which involve methylation and acetylation, as well as post-transcriptional regulation by noncoding RNAs [41,129]. It is not within the scope of this review to explain all these mechanisms in detail, but the role of some epigenetic processes should be elucidated to understand the defects in enamel formation described in Section 6.1. The abovementioned regulatory mechanisms modulate the timing and level of gene expression required for proper enamel matrix secretion [129]. Disruptions of normal epigenetic patterns may impair enamel formation even when the genetic code is intact. A recent study revealed that deleting the epigenetic regulator KMT2D in the enamel organ of mice causes severe enamel hypoplasia and hypomineralization [17]. Other factors influencing epigenetic control are environmental influences, such as nutritional deficiencies or exposure to certain chemicals during tooth development, which have been linked to altered enamel formation. One well-documented example is exposure to fluoride, which leads to hypermethylation and disrupted ameloblast activity by activating histone acetyltransferases. Another example is the influence of bisphenol-A on the development of enamel formation, which also causes rat teeth to become hypomineralized due to increased expression of enamelin and reduced expression of KLK4 [129,130].

Following the narrative of a recent review by Bomfim et al., the potential clinical application of genetic testing to optimize patient care now appears both feasible and within reach [131].

##### Fluorosis: How Fluoride Affects Tooth Development

**Figure 12:**
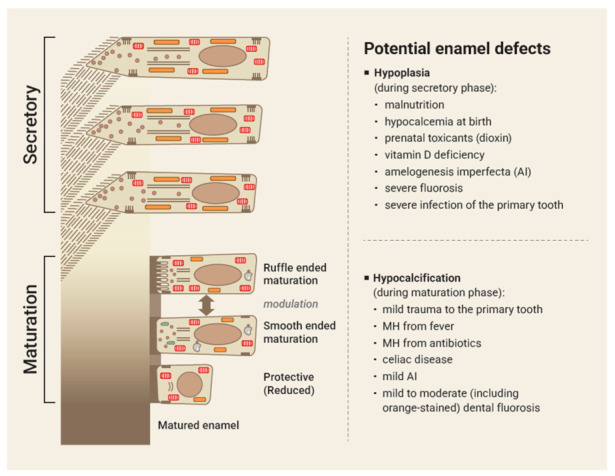
**Left**: Overview of the changes during the two main processes: secretion and maturation. In particular, functional changes in the organelles within the ameloblast cell body are depicted here. The color gradient from top to bottom indicates the degree of ripening of the enamel until it matures. **Right**: typical enamel malformations occurring during different stages of amelogenesis. The upper layer is during the secretory stage, and the lower layer is during the maturation stage.

The severity of dental fluorosis can range from barely noticeable to the naked eye to severe, where the teeth are mostly chalky white with heavy brown stain and the enamel is pitted or completely missing (Figure 12) [132]. Fluoride has been shown to inhibit many enzymes, especially serine proteinases, which are responsible for the degradation of enamel proteins [133]. The greater the fluoride content, the greater the amount of fluorapatite, the more stable the crystal, and the more rapid the crystal growth. This traps proteins, resulting in porous enamel [134], which in turn lowers the pH of the mineralizing environment, which also affects enzyme removal of the protein [135]. More recently, many molecular biological changes within enamel formation cells resulting from excess fluoride exposure have been investigated. Fluoride mediates the MAPK signalling pathway, which can lead to changes in gene expression, cell stress, and cell death [136]. Fluoride regulates epigenetic treatment via H3 acetylation [137]. High-fluoride-induced Foxo1 expression is a cause of low KLK4 expression [138]. Fluoride-mediated ameloblast apoptosis occurs via two vital pathways: the mitochondria-mediated pathway and the endoplasmic reticulum pathway [139].

The main reason for the development of fluorosis is chronic exposure to fluoride from birth to eight years of age [140]. This time frame overlaps with the time of development of the secondary dentition, as described above [141]. Therefore, it is assumed that the condition of fluorosis might be correlated with the use of fluoridation on the primary dentition starting with the first tooth erupting [141]. Warren et al. were accordingly able to show that fluorosis in the primary dentition is rare [142]. From a chemical point of view, fluoride readily reacts with calcium ions, forming insoluble calcium fluoride. This may also negatively impact natural HAP crystal formation.

##### Enamel Hypomineralization

Enamel hypomineralization was recognized as a developmental defect of enamel (DDE) more than a century ago [143,144,145]. It is characterized by demarcated opacities that range in color (in order of severity) from chalky white to cream to brown (Figure 12). In these lesions, the enamel is mechanically weak, crumbles easily and predisposes affected areas to posteruptive breakdown and decay. The global prevalence of hypomineralization is approximately 20%, reaching or even surpassing that of dental caries [146]. Children between the ages of 5 and 12 years are most frequently affected. Unlike that of dental caries, the etiology of hypomineralization has remained unclear.

The teeth most frequently affected by these hypomineralization defects are the first permanent molars (tooth number 6, six-year-old molars), followed by the second permanent molars, first deciduous molars and incisors. However, case reports indicate that hypomineralization can occur in practically all teeth [147]. Unfortunately, reports of hypomineralized enamel defects in different tooth types, global locations and demographic settings have led to more than 20 different names or acronyms for the same condition, along with variable diagnosis, grading and treatment guides. A unifying nomenclature was proposed by the D3 Group and recently adopted by the journal *Pediatric Dentistry* [148]. Until such terminology issues are resolved, “chalky teeth” is a very useful, public-friendly term to describe this condition regardless of which teeth are affected and to engage young patients and their caregivers.

Considering that the majority of enamel minerals are incorporated into the tissue during the maturation stage, it is reasonable to conclude that hypomineralization is a defect that occurs during this stage of enamel formation. This has been supported by microstructural analyses and the observation that albumin, a serum protein not normally found in the enamel matrix, is present in affected areas of hypomineralized enamel [145]. The cleavage of albumin appears to be far less efficient than that of enamel proteins, which has led to the “mineral-poisoning” model [143], offering a significant advance towards understanding the pathology of this condition. However, how albumin can enter the enamel space is not well understood. The enamel in the most frequently affected first permanent molars (“six-year molars”) matures during a time window roughly covering the first 18 months of life, so adverse events leading to chalky six-year molars occur during that period. Identifying these adverse events and delineating them from contributing (epi-)genetic factors is a major challenge but also a great opportunity to develop preventive strategies. Thus, for this medical problem with a dental manifestation, concerted efforts by researchers, pediatricians and dentists are urgently needed.

##### Enamel Hypoplasia

The first report of enamel hypoplasia dates back to 1912 [149]. As mentioned earlier, the growth of enamel occurs during the secretory stage, so enamel hypoplasia is likely a disturbance related to production, secretion, proper processing of EMPs, or mutations in the encoding genes that lead to such disturbances (Figure 12) [150]. Among the main EMPs, AMEL and ENAM knockout in mice are hallmarks of enamel hypoplasia with structural anomalies. The list of genes that are mutated in hypoplastic enamel phenotypes (syndromic or nonsyndromic) is long and growing and includes many more genes than those coding for EMPs [151]. From this observation, it can be deduced that the enamel organ is particularly sensitive to the dysregulation of biochemical and cell signalling pathways that operate in multiple tissues.

### 6.2. What Can We Learn from Nature?

Biomimetic strategies in restorative dentistry and oral care

Since enamel is a tissue with unique properties, research has been performed to mimic this biomineral as a tooth restoration material [152]. Compared with enamel, traditional tooth restorative materials such as ceramics or polymers have different hardness values. Moreover, these synthetic materials cannot be remineralized with saliva. Many papers have described the production of a “synthetic enamel” for use as a potential tooth restorative material [153,154,155,156].

In addition to promising tooth restorative materials inspired by enamel, prevention is also an important issue. Toothpaste formulations have undergone significant improvements over the last few decades, especially due to the use of biomimetic approaches [157].

As described in the previous sections, natural enamel formation involves a reaction process that begins with calcium and phosphate ions forming ACPs, which finally transform into crystalline HAP. This is why all of these ions (i.e., calcium, phosphate) and molecules (i.e., ACP, HAP) are used in oral care formulations for the biomimetic remineralization of enamel.

Particulate HAP is commonly used in oral care products such as toothpastes and mouthwashes [158]. Various studies have demonstrated its ability to remineralize both enamel [159,160] and dentin [161]. It has also been shown to remineralize hypomineralization-affected teeth [162] and provides efficient caries prevention [163]. ACP is typically used in combination with casein phosphopeptide (CPP-ACP), which stabilizes ACP and prevents its agglomeration. CPP-ACP was also shown to promote enamel remineralization [164]. In the 2025 IADR/PER General Session in Barcelona, calcium hypophosphite was described for the first time as a promising new active ingredient for oral care. It is an efficient source for calcium ions, which contribute to remineralization [165,166,167]. Moreover, when in contact with saliva, calcium hypophosphite directly forms crystalline hydroxyapatite, which occludes open dentin tubules—a prerequisite for relief from dentin hypersensitivity [166,167]. Additionally, in the field of biomimetic enamel remineralization, self-assembling peptides such as peptide P11-4 (consisting of 11 amino acids) have been shown to support enamel remineralization [168].

Another recent study demonstrated the biomimetic potential of keratin by successfully replicating natural enamel formation processes through self-assembled β-sheet architectures that direct organized mineral growth, restoring both the mechanical integrity and the crystalline structure of damaged enamel. The research exemplifies how nature-inspired approaches can yield clinically viable, sustainable solutions, with the simple fabrication process and broad applicability of keratin, positioning it as a promising platform for advancing biomimetic strategies across dental and biomedical fields [169].

## 7. Summary

This review provides a comprehensive synthesis of contemporary knowledge on human enamel formation (amelogenesis), integrating embryology, cellular differentiation, molecular regulation, structural organization, and clinically relevant disturbances. Tooth enamel is produced by a highly specialized epithelial cell lineage (ameloblasts) that emerges from reciprocal epithelial–mesenchymal signalling during odontogenesis. Enamel formation proceeds through well-defined stages: prescretory, secretory, transition, and maturation, each characterized by distinct ameloblast morphologies and functions. Together, these stages orchestrate the production of an organic matrix, its controlled proteolytic processing, and the sequential deposition and crystallization of calcium-phosphate phases that yield mature hydroxyapatite (HAP) with a hierarchical rod/interrod architecture.

At the molecular level, enamel morphogenesis is directed by a relatively small set of enamel matrix proteins and proteases whose temporal and spatial regulation is critical. Amelogenin, ameloblastin, and enamelin constitute the major structural proteins that scaffold initial mineral nucleation and guide nanocluster assembly into oriented crystallites; MMP20 and KLK4 perform the stage-specific proteolytic processing required to permit crystal growth and organic matrix removal. Post-translational modifications (for example, phosphorylation of specific residues) and a cohort of additional secreted factors (e.g., AMTN, ODAM and ODAPH) further fine-tune surface mineralization, cell–mineral attachment and the formation of the enamel surface layer. Concomitantly, ameloblasts coordinate a complex set of ion transporters, channels, and pH-regulatory mechanisms and cyclically alternate apical membrane phenotypes (ruffled vs. smooth ended) to deliver Ca^2+^, PO43- and HCO3- to buffer proton generation and maintain the ionic milieu required for ordered HAP crystallization.

The physical structure of enamel, from nanometer-scale crystallites to millimeter-scale rod/interrod patterns and incremental growth lines (cross-striations, Striae of Retzius, neonatal line), emerges from these biological controls. Subtle orientations and controlled misalignments of crystallites confer toughness through crack deflection and energy dissipation, whereas the progressive reduction in organic content (from a protein-rich early matrix to ~97% mineral in mature enamel) underlies the extreme hardness and wear resistance of the tissue.

Clinically important enamel pathologies arise when any element of this tightly regulated process is perturbed. Genetic mutations in AMELX, ENAM, AMBN, MMP20, KLK4 and other genes produce heterogeneous phenotypes grouped under amelogenesis imperfecta (AI), ranging from hypoplastic to hypomineralized or mixed presentations. Environmental agents (notably excess fluoride) alter enamel protein processing, cell signalling, and epigenetic marks, resulting in fluorosis with characteristic surface and subsurface changes. One example to keep in mind is the effect of fluoridation during critical developmental stages of primary and secondary dentition, resulting in potential fluorosis in the secondary dentition. Developmental hypomineralization (including conditions commonly termed MH or “chalky teeth”) appears to result primarily from disturbances during the maturation window and is associated with incorporation of non-matrix proteins (e.g., serum albumin) and impaired protein removal, yielding mechanically weak, post-eruptively vulnerable enamel. Hypoplasia reflects disruptions of the secretory stage and results in quantitatively reduced enamel. Epigenetic regulators and environmentally induced epigenomic changes modulate the expression timing and levels of critical amelogenesis genes, providing an explanatory bridge between genetic susceptibility and environmental triggers.

From a translational perspective, the mechanistic insights summarized here directly inform both preventive and restorative strategies. Recognition of vulnerable developmental time windows emphasizes targeted public health and clinical prevention (for example, fluoride exposure control during tooth formation and timely surveillance of first permanent molars). Biomimetic approaches to remineralization take direct inspiration from the natural ACP → HAP pathway and include particulate hydroxyapatite formulations, stabilized amorphous calcium phosphate (CPP-ACP), self-assembling peptides that promote mineral growth (e.g., P11-4), novel calcium donors (e.g., calcium hypophosphite), and emerging organic scaffolds (such as keratin-based templates) that guide organized mineral deposition. These strategies offer promising, minimally invasive options to restore mineral density, occlude dentinal tubules, and mitigate sensitivity. These findings underscore the clinical value of translating fundamental enamel biology into pragmatic oral-care interventions.

### Strengths and Limitations

The strengths of this review lie in its integration of cellular, molecular, structural, and clinical perspectives to produce a practical primer for clinicians while highlighting pathways amenable to therapeutic exploitation. Limitations include the scoping nature of the systematic search (which deliberately excluded many primary defect-focused studies from the systematic arm and instead addressed them narratively) and the incomplete mechanistic resolution of certain maturation-stage processes (for example, precise routes for removal of peptide fragments and the detailed orchestration of ion transport at the nanoscale). A formal risk-of-bias assessment was not conducted; however, potential bias was mitigated through the involvement of three independent authors. Given the complexity of the subject matter, the review was deliberately limited to secondary literature, including narrative reviews, systematic reviews, and meta-analyses, with primary studies not being considered. Furthermore, the direct investigation of certain physiological aspects in humans presents considerable methodological and ethical challenges; consequently, the majority of the underlying evidence is derived from rodent models.

## 8. Conclusions

This review summarizes our current knowledge on the complex mechanism of tooth enamel formation for a clinical target audience. Ameloblasts pass the presecretory, secretory, transition and maturation phases, in which an organic matrix (AMEL, AMBN, ENAM) is secreted, proteolytically processed (MMP20, KLK4), and stepwise replaced with HAP. Ion transport mechanisms and pH regulation ensure the ordered organization of crystallites into rod and interrod structures.

Disturbances in genes, signalling pathways or the cellular microenvironment cause typical defects: hypoplasia (secretory phase), hypomineralization (especially the maturation phase, including the incorporation of albumin), fluorosis (overfluoridation with altered protein processing, signal transduction and epigenetics) and heterogeneous AI phenotypes (e.g., mutations in AMELX, ENAM, AMBN, MMP20, and KLK4). Understanding the temporal ‘vulnerability windows’ is central to prevention and early diagnosis.

Biomimetic approaches translate biological principles into clinical strategies: remineralizing agents such as particulate hydroxyapatite, CPP-ACP, self-assembling peptides (e.g., P11-4), and new calcium sources (e.g., calcium hypophosphite) support ordered mineral growth. We hope that the information provided herein will be useful for practicing dentists and other oral healthcare providers to correlate some of the clinical observations with their scientific foundations.

## Data Availability

No new data were created or analyzed in this study. Data sharing is not applicable to this article.

## Supplementary Materials

The following supporting information can be downloaded at: https://www.mdpi.com/article/10.3390/dj14070421/s1, Table S1: Results literature search; Table S2: Preferred Reporting Items for Systematic reviews and Meta-Analyses extension for Scoping Reviews (PRISMA-ScR) Checklist.

## Abbreviations

The following abbreviations are used in this manuscript:

MH	Molar hypomineralization
MIH	Molar incisor hypomineralization
HAP	hydroxyapatite
PRISMA	Preferred reporting items for systematic reviews and meta-analyses
Wnts	Wingless-Int proteins
BMPs	Bone morphogenic proteins
FGFs	Fibroblast growth factors
SHH	Sonic hedgehog protein
EDAR	Ectodysplasin A receptor
DEJ	Dentin-enamel junction
SEM	Scanning electron microscopy
rER	Rough endoplasmic reticulum
EMPs	Enamel matrix proteins
RA	Ruffling ended Ameloblast
SA	Smooth ended Ameloblast
KLK4	Kallikrein-related peptidase 4
MMP20	Metalloproteinase 20
AMEL	Amelogenin
ACP	Amorphous calcium phosphate
AMBN	Ameloblastin
ENAM	Enamelin
TUFT1	Tuftelin
AI	Amelogenesis imperfecta
FAM20C	Family with sequence similarity 20, member C
FAM38H	Family with sequence similarity 83 member H
SCPPs	Secreted calcium-binding phosphoproteins
AMTN	Amelotin
ODAM	Odontogenic-ameloblast associated protein
ODAPH	Odontogenesis-associated phosphoprotein
SCPPPQ	Secreted calcium-binding phosphoprotein rich in proline and glutamine
FDC-SP	Follicular dendritic cell-secreted protein

## References

[1] Wu Y.-Q., Arsecularatne J.A., Hoffman M. (2017). Attrition-corrosion of human dental enamel: A review. Biosurface Biotribology.

[2] Hubbard M.J., Kon J.C. (2002). Proteomic analysis of dental tissues. J. Chromatogr. B Anal. Technol. Biomed. Life Sci..

[3] WHO (2025). Highlights Oral Health Neglect Affecting Nearly Half of the World’s Population. https://www.who.int/news/item/18-11-2022-who-highlights-oral-health-neglect-affecting-nearly-half-of-the-world-s-population.

[4] Lopes L.B., Machado V., Mascarenhas P., Mendes J.J., Botelho J. (2021). The prevalence of molar-incisor hypomineralization: A systematic review and meta-analysis. Sci. Rep..

[5] Niazi F.C., Pepper T., Niazi F.C., Pepper T. (2023). StatPearls: Dental Fluorosis. StatPearls [Internet].

[6] Bawden J.W. (1989). Calcium transport during mineralization. Anat. Rec..

[7] Bartlett J.D., Simmer J.P. (2014). Kallikrein-related peptidase-4 (KLK4): Role in enamel formation and revelations from ablated mice. Front. Physiol..

[8] Shaw W.J., Tarasevich B.J., Buchko G.W., Arachchige R.M.J., Burton S.D. (2020). Controls of nature: Secondary, tertiary, and quaternary structure of the enamel protein amelogenin in solution and on hydroxyapatite. J. Struct. Biol..

[9] Deutsch D., Catalano-Sherman J., Dafni L., David S., Palmon A. (1995). Enamel matrix proteins and ameloblast biology. Connect. Tissue Res..

[10] Goldberg M., Septier D., Lecolle S., Chardin H., A Quintana M., Acevedo A.C., Gafni G., Dillouya D., Vermelin L., Thonemann B. (1995). Dental mineralization. Int. J. Dev. Biol..

[11] Simmer J.P., Hu J.C. (2001). Dental enamel formation and its impact on clinical dentistry. J. Dent. Educ..

[12] Gruenbaum-Cohen Y., Tucker A.S., Haze A., Shilo D., Taylor A.L., Shay B., Sharpe P.T., Mitsiadis T.A., Ornoy A., Blumenfeld A. (2009). Amelogenin in cranio-facial development: The tooth as a model to study the role of amelogenin during embryogenesis. J. Exp. Zool. B Mol. Dev. Evol..

[13] Sabel N. (2012). Enamel or primary teeth—Morphological and chemical aspects. Swed. Dent. J..

[14] Lu Y., Papagerakis P., Yamakoshi Y., Hu J.C.-C., Bartlett J.D., Simmer J.P. (2008). Functions of KLK4 and MMP-20 in dental enamel formation. Biol. Chem..

[15] Bartlett J.D. (2013). Dental enamel development: Proteinases and their enamel matrix substrates. ISRN Dent..

[16] Kegulian N.C., Visakan G., Bapat R.A., Moradian-Oldak J. (2024). Ameloblastin and its multifunctionality in amelogenesis: A review. Matrix Biol..

[17] Duverger O., Lee J.S. (2024). The intricacies of tooth enamel: Embryonic origin, development and human genetics. J. Struct. Biol..

[18] Robinson C., Briggs H.D., Atkinson P.J., Weatherell J.A. (1979). Matrix and mineral changes in developing enamel. J. Dent. Res..

[19] Smith C.E. (1998). Cellular and chemical events during enamel maturation. Crit. Rev. Oral Biol. Med..

[20] Warshawsky H. (1989). Organization of crystals in enamel. Anat. Rec..

[21] Kallenbach E. (1967). Cell architecture in the papillary layer of rat incisor enamel organ at the stage of enamel maturation. Anat. Rec..

[22] Gil-Bona A., Bidlack F.B. (2020). Tooth Enamel and its Dynamic Protein Matrix. Int. J. Mol. Sci..

[23] Enax J., Fandrich P., zur Schulze Wiesche E., Epple M. (2024). The Remineralization of Enamel from Saliva: A Chemical Perspective. Dent. J..

[24] Wilmers J., Bargmann S. (2020). Nature’s design solutions in dental enamel: Uniting high strength and extreme damage resistance. Acta Biomater..

[25] Ganss B., Abbarin N. (2014). Maturation and beyond: Proteins in the developmental continuum from enamel epithelium to junctional epithelium. Front. Physiol..

[26] Tricco A.C., Lillie E., Zarin W., O’Brien K.K., Colquhoun H., Levac D., Moher D., Peters M.D.J., Horsley T., Weeks L. (2018). PRISMA Extension for Scoping Reviews (PRISMA-ScR): Checklist and Explanation. Ann. Intern. Med..

[27] Lacruz R.S., Habelitz S., Wright J.T., Paine M.L. (2017). Dental enamel formation and implications for oral health and disease. Physiol. Rev..

[28] Moradian-Oldak J. (2012). Protein-mediated enamel mineralization. Front. Biosci..

[29] Pandya M., Diekwisch T.G.H. (2021). Amelogenesis: Transformation of a protein-mineral matrix into tooth enamel. J. Struct. Biol..

[30] Simmer J.P., Fincham A.G. (1995). Molecular Mechanisms of Dental Enamel Formation. Crit. Rev. Oral Biol. Med..

[31] Moll K.-J., Moll M. (2003). Anatomie: Kurzlehrbuch zum Gegenstandskatalog.

[32] Hutami I.R., Arinawati D.Y., Rahadian A., Dewi R.C., Rochmah Y.S., Christiono S., Afroz S. (2025). Roles of calcium in ameloblasts during tooth development: A scoping review. J. Taibah Univ. Med. Sci..

[33] Nanci A. (2017). Ten Cate’s Oral Histology—E-Book: Development, Structure, and Function.

[34] Spemann H., Mangold H. (1924). Induction of embryonic primordia by implantation of organizers from a different species. Roux’s Arch. Entw. Mech..

[35] Zeichner-David M., Diekwisch T., Fincham A., Lau E., MacDougall M., Moradian-Oldak J., Simmer J., Snead M., Slavkin H.C. (1995). Control of ameloblast differentiation. Int. J. Dev. Biol..

[36] Balic A., Thesleff I. (2015). Tissue Interactions Regulating Tooth Development and Renewal. Curr. Top. Dev. Biol..

[37] Simmer J., Papagerakis P., Smith C., Fisher D., Rountrey A., Zheng L., Hu J.-C. (2010). Regulation of dental enamel shape and hardness. J. Dent. Res..

[38] Matalová E., Lungová V., Sharpe P., Vishwakarma A., Sharpe P., Shi S., Ramalingam M. (2015). Development of Tooth and Associated Structures. Stem Cell Biology and Tissue Engineering in Dental Sciences.

[39] Jin Y., Wang C., Cheng S., Zhao Z., Li J. (2017). MicroRNA control of tooth formation and eruption. Arch. Oral Biol..

[40] Zhou Y., Zheng L., Sun J., Ye L., Zhou X., Gao B. (2015). Expression and Function of MicroRNAs in Enamel Development. Curr. Stem Cell Res. Ther..

[41] Fan Y., Zhou Y., Zhou X., Xu X., Pi C., Xu R., Zheng L. (2015). Epigenetic Control of Gene Function in Enamel Development. Curr. Stem Cell Res. Ther..

[42] Thesleff I., Hurmerinta K. (1981). Tissue interactions in tooth development. Differentiation.

[43] Slavkin H.C. (1989). Positional signalling and patterning for amelogenesis in mouse molar tooth development. Connect. Tissue Res..

[44] Sharpe P.T. (1995). Homeobox genes and orofacial development. Connect. Tissue Res..

[45] Baranova J., Büchner D., Götz W., Schulze M., Tobiasch E. (2020). Tooth Formation: Are the Hardest Tissues of Human Body Hard to Regenerate?. Int. J. Mol. Sci..

[46] Karimian A., Ahmadi Y., Yousefi B. (2016). Multiple functions of p21 in cell cycle, apoptosis and transcriptional regulation after DNA damage. DNA Repair.

[47] Slavkin H.C. (1991). Molecular determinants during dental morphogenesis and cytodifferentiation: A review. J. Craniofac. Genet. Dev. Biol..

[48] Vaahtokari A., Aberg T., Jernvall J., Keränen S., Thesleff I. (1996). The enamel knot as a signaling center in the developing mouse tooth. Mech. Dev..

[49] Jernvall J., Thesleff I. (2000). Reiterative signaling and patterning during mammalian tooth morphogenesis. Mech. Dev..

[50] (2025). Tooth Development 7|Digital Histology. https://digitalhistology.org/organs-systems/digestive/oral-cavity/tooth/tooth-development/tooth-development-8/.

[51] Brodusch N., Brahimi S.V., De Melo E.B., Song J., Yue S., Piché N., Gauvin R. (2021). Scanning Electron Microscopy versus Transmission Electron Microscopy for Material Characterization: A Comparative Study on High-Strength Steels. Scanning.

[52] Martin L.B., Boyde A., Grine F.E. (1988). Enamel structure in primates: A review of scanning electron microscope studies. Scanning Microsc..

[53] Nylen M.U. (1964). Electron Microscope and Allied Biophysical Approaches to the Study of Enamel Mineralization. J. R. Microsc. Soc..

[54] Fabritius H., Enax J., Meyer F. (2021). Eine Reise ins Innere unserer Zähne. 2. Auflage.

[55] Kardos T.B. (1998). Enamel—An overview. N. Z. Dent. J..

[56] Ali S., Farooq I. (2019). A Review of the Role of Amelogenin Protein in Enamel Formation and Novel Experimental Techniques to Study its Function. Protein Pept. Lett..

[57] Lacruz R.S., Nanci A., Kurtz I., Wright J.T., Paine M.L. (2010). Regulation of pH During Amelogenesis. Calcif. Tissue Int..

[58] Varga G., Kerémi B., Bori E., Földes A. (2015). Function and repair of dental enamel—Potential role of epithelial transport processes of ameloblasts. Pancreatology.

[59] Pham C.-D., Smith C.E., Hu Y., Hu J.C.-C., Simmer J.P., Chun Y.-H.P. (2017). Endocytosis and Enamel Formation. Front. Physiol..

[60] Moradian-Oldak J., George A. (2021). Biomineralization of Enamel and Dentin Mediated by Matrix Proteins. J. Dent. Res..

[61] Sasaki T., Takagi M., Yanagisawa T. (1997). Structure and function of secretory ameloblasts in enamel formation. Ciba Found. Symp..

[62] Robinson C., Kirkham J., Brookes S.J., Bonass W.A., Shore R.C. (1995). The chemistry of enamel development. Int. J. Dev. Biol..

[63] Yin K., Paine M.L. (2017). Bicarbonate Transport During Enamel Maturation. Calcif. Tissue Int..

[64] Bartlett J.D., Simmer J.P. (1999). Proteinases in developing dental enamel. Crit. Rev. Oral Biol. Med..

[65] Aoba T. (1996). Recent observations on enamel crystal formation during mammalian amelogenesis. Anat. Rec..

[66] Moradian-Oldak J. (2001). Amelogenins: Assembly, processing and control of crystal morphology. Matrix Biol..

[67] Wu K., Li X., Bai Y., Heng B.C., Zhang X., Deng X. (2024). The circadian clock in enamel development. Int. J. Oral Sci..

[68] Lacruz R.S., Smith C.E., Kurtz I., Hubbard M.J., Paine M.L. (2013). New paradigms on the transport functions of maturation-stage ameloblasts. J. Dent. Res..

[69] Robinson C., Brookes S.J., Bonass W.A., Shore R.C., Kirkham J. (1997). Enamel maturation. Ciba Found. Symp..

[70] Smith C.E., McKee M.D., Nanci A. (1987). Cyclic induction and rapid movement of sequential waves of new smooth-ended ameloblast modulation bands in rat incisors as visualized by polychrome fluorescent labeling and GBHA-staining of maturing enamel. Adv. Dent. Res..

[71] Habelitz S. (2015). Materials engineering by ameloblasts. J. Dent. Res..

[72] Simmer J.P., Richardson A.S., Hu Y.-Y., Smith C.E., Ching-Chun Hu J. (2012). A post-classical theory of enamel biomineralization… and why we need one. Int. J. Oral Sci..

[73] Margolis H.C., Beniash E., Fowler C.E. (2006). Role of macromolecular assembly of enamel matrix proteins in enamel formation: 8. J. Dent. Res..

[74] Dorozhkin S.V., Epple M. (2002). Biological and Medical Significance of Calcium Phosphates. Angew. Chem. Int. Ed..

[75] Habelitz S., Bai Y. (2021). Mechanisms of Enamel Mineralization Guided by Amelogenin Nanoribbons. J. Dent. Res..

[76] Ang S.F., Saadatmand M., Swain M.V., Klocke A., Schneider G.A. (2012). Comparison of mechanical behaviors of enamel rod and interrod regions in enamel. J. Mater. Res..

[77] Beniash E., Metzler R.A., Lam R.S.K., Gilbert P.U.P.A. (2009). Transient amorphous calcium phosphate in forming enamel. J. Struct. Biol..

[78] Beniash E., Stifler C.A., Sun C.-Y., Jung G.S., Qin Z., Buehler M.J., Gilbert P.U.P.A. (2019). The hidden structure of human enamel. Nat. Commun..

[79] Moradian-Oldak J. (2007). The emergence of “nanospheres” as basic structural components adopted by amelogenin. J. Dent. Res..

[80] Sire J.-Y., Davit-Béal T., Delgado S., Gu X. (2007). The origin and evolution of enamel mineralization genes. Cells Tissues Organs.

[81] Wright J.T. (2023). Enamel Phenotypes: Genetic and Environmental Determinants. Genes.

[82] Fincham A.G., Moradian-Oldak J., Simmer J.P. (1999). The structural biology of the developing dental enamel matrix. J. Struct. Biol..

[83] Fincham A.G., Moradianoldak J., Diekwisch T.G., Lyaruu D.M., Wright J.T., Bringas P., Slavkin H.C. (1995). Evidence for amelogenin “nanospheres” as functional components of secretory-stage enamel matrix. J. Struct. Biol..

[84] Fincham A.G., Simmer J.P. (1997). Amelogenin proteins of developing dental enamel. Ciba Found. Symp..

[85] He X., Wu S., Martinez-Avila O., Cheng Y., Habelitz S. (2011). Self-aligning amelogenin nanoribbons in oil-water system. J. Struct. Biol..

[86] Wald T., Spoutil F., Osickova A., Prochazkova M., Benada O., Kasparek P., Bumba L., Klein O.D., Sedlacek R., Sebo P. (2017). Intrinsically disordered proteins drive enamel formation via an evolutionarily conserved self-assembly motif. Proc. Natl. Acad. Sci. USA.

[87] Buchko G.W., Mergelsberg S.T., Tarasevich B.J., Shaw W.J. (2022). Residue-Specific Insights into the Intermolecular Protein-Protein Interfaces Driving Amelogenin Self-Assembly in Solution. Biochemistry.

[88] Fukumoto S., Yamada A., Nonaka K., Yamada Y. (2005). Essential roles of ameloblastin in maintaining ameloblast differentiation and enamel formation. Cells Tissues Organs.

[89] Siddiqui S., Al-Jawad M. (2016). Enamelin Directs Crystallite Organization at the Enamel-Dentine Junction. J. Dent. Res..

[90] Hu J.C.-C., Hu Y., Lu Y., Smith C.E., Lertlam R., Wright J.T., Suggs C., McKee M.D., Beniash E., Kabir M.E. (2014). Enamelin is critical for ameloblast integrity and enamel ultrastructure formation. PLoS ONE.

[91] Hu J.C.-C., Yamakoshi Y. (2003). Enamelin and autosomal-dominant amelogenesis imperfecta. Crit. Rev. Oral Biol. Med..

[92] Hart T.C., Hart P.S., Gorry M.C., Michalec M.D., Ryu O.H., Uygur C., Ozdemir D., Firatli S., Aren G., Firatli E. (2003). Novel ENAM mutation responsible for autosomal recessive amelogenesis imperfecta and localised enamel defects. J. Med. Genet..

[93] Kim J.-W., Seymen F., Lin B.P.-J., Kızıltan B., Gencay K., Simmer J.P., Hu J.-C. (2005). ENAM mutations in autosomal-dominant amelogenesis imperfecta. J. Dent. Res..

[94] Wang Y.-L., Lin H.-C., Liang T., Lin J.-Y., Simmer J., Hu J.-C., Wang S.-K. (2024). ENAM Mutations Can Cause Hypomaturation Amelogenesis Imperfecta. J. Dent. Res..

[95] Deutsch D., Palmon A., Dafni L., Catalano-Sherman J., Young M.F., Fisher L.W. (1995). The enamelin (tuftelin) gene. Int. J. Dev. Biol..

[96] Deutsch D., Dafni L., Palmon A., Hekmati M., Young M.F., Fisher L.W. (1997). Tuftelin: Enamel mineralization and amelogenesis imperfecta. Ciba Found. Symp..

[97] Jackson A., Moss C., E Chandler K., Balboa P.L., Bageta M.L., Petrof G., E Martinez A., Liu L., Guy A., E Mellerio J. (2023). Biallelic TUFT1 variants cause woolly hair, superficial skin fragility and desmosomal defects. Br. J. Dermatol..

[98] Simmer J.P., Hu J.C.C. (2002). Expression, structure, and function of enamel proteinases. Connect. Tissue Res..

[99] Moradian-Oldak J., Gharakhanian N., Jimenez I. (2002). Limited Proteolysis of Amelogenin: Toward Understanding the Proteolytic Processes in Enamel Extracellular Matrix. Connect. Tissue Res..

[100] Sun Z., Fan D., Fan Y., Du C., Moradian-Oldak J. (2008). Enamel proteases reduce amelogenin-apatite binding. J. Dent. Res..

[101] Uskoković V., Khan F., Liu H., Witkowska H.E., Zhu L., Li W., Habelitz S. (2011). Hydrolysis of amelogenin by matrix metalloprotease-20 accelerates mineralization in vitro. Arch. Oral Biol..

[102] Iwata T., Yamakoshi Y., Hu J.-C., Ishikawa I., Bartlett J., Krebsbach P., Simmer J. (2007). Processing of ameloblastin by MMP-20. J. Dent. Res..

[103] Chun Y.-H., Yamakoshi Y., Yamakoshi F., Fukae M., Hu J.-C., Bartlett J., Simmer J. (2010). Cleavage site specificity of MMP-20 for secretory-stage ameloblastin. J. Dent. Res..

[104] Yamakoshi Y., Hu J.C.-C., Fukae M., Yamakoshi F., Simmer J.P. (2006). How do enamelysin and kallikrein 4 process the 32-kDa enamelin?. Eur. J. Oral Sci..

[105] Gabe C.M., Bui A.T., Lukashova L., Verdelis K., Vasquez B., Beniash E., Margolis H.C. (2024). Role of amelogenin phosphorylation in regulating dental enamel formation. Matrix Biol..

[106] Cui J., Xiao J., Tagliabracci V.S., Wen J., Rahdar M., Dixon J.E. (2015). A secretory kinase complex regulates extracellular protein phosphorylation. Elife.

[107] Yan W.-J., Ma P., Tian Y., Wang J.-Y., Qin C.-L., Feng J.Q., Wang X.-F. (2017). The importance of a potential phosphorylation site in enamelin on enamel formation. Int. J. Oral Sci..

[108] Mu H., Dong Z., Wang Y., Chu Q., Gao Y., Wang A., Wang Y., Liu X., Gao Y. (2022). Odontogenesis-Associated Phosphoprotein (ODAPH) Overexpression in Ameloblasts Disrupts Enamel Formation via Inducing Abnormal Mineralization of Enamel in Secretory Stage. Calcif. Tissue Int..

[109] Chan H.-C., Mai L., Oikonomopoulou A., Richardson A., Wang S.-K., Simmer J., Hu J.-C. (2010). Altered enamelin phosphorylation site causes amelogenesis imperfecta. J. Dent. Res..

[110] Wang X., Jung J., Liu Y., Yuan B., Lu Y., Feng J., Qin C. (2013). The specific role of FAM20C in amelogenesis. J. Dent. Res..

[111] Wang S., Zhang H., Hu C., Liu J., Chadha S., Kim J., Simmer J., Hu J. (2021). FAM83H and Autosomal Dominant Hypocalcified Amelogenesis Imperfecta. J. Dent. Res..

[112] Kim H.-E., Hong J.H. (2018). The overview of channels, transporters, and calcium signaling molecules during amelogenesis. Arch. Oral Biol..

[113] Bronckers A.L.J.J. (2017). Ion Transport by Ameloblasts during Amelogenesis. J. Dent. Res..

[114] Duan X. (2014). Ion channels, channelopathies, and tooth formation. J. Dent. Res..

[115] Frank R.M. (1979). Electron microscope autoradiography of calcified tissues. Int. Rev. Cytol..

[116] Varga G., DenBesten P., Rácz R., Zsembery Á. (2018). Importance of bicarbonate transport in pH control during amelogenesis—Need for functional studies. Oral Dis..

[117] Abbarin N., San Miguel S., Holcroft J., Iwasaki K., Ganss B. (2015). The enamel protein amelotin is a promoter of hydroxyapatite mineralization. J. Bone Miner. Res..

[118] Nakayama Y., Holcroft J., Ganss B. (2015). Enamel Hypomineralization and Structural Defects in Amelotin-deficient Mice. J. Dent. Res..

[119] Wazen R.M., Moffatt P., Ponce K.J., Kuroda S., Nishio C., Nanci A. (2015). Inactivation of the Odontogenic ameloblast-associated gene affects the integrity of the junctional epithelium and gingival healing. Eur. Cells Mater..

[120] Fouillen A., Neves J.D.S., Mary C., Castonguay J.-D., Moffatt P., Baron C., Nanci A. (2017). Interactions of AMTN, ODAM and SCPPPQ1 proteins of a specialized basal lamina that attaches epithelial cells to tooth mineral. Sci. Rep..

[121] Nouri S., Holcroft J., Caruso L.-L., Vuong T.V., Simmons C.A., Master E.R., Ganss B. (2022). An SCPPPQ1/LAM332 protein complex enhances the adhesion and migration of oral epithelial cells: Implications for dentogingival regeneration. Acta Biomater..

[122] Masood F., Benavides E. (2018). Alterations in Tooth Structure and Associated Systemic Conditions. Radiol. Clin. N. Am..

[123] Simmer J.P., Hu J.C.-C., Hu Y., Zhang S., Liang T., Wang S.-K., Kim J.-W., Yamakoshi Y., Chun Y.-H., Bartlett J.D. (2021). A genetic model for the secretory stage of dental enamel formation. J. Struct. Biol..

[124] Sofaer J.A. (1975). Genetic variation and tooth development. Br. Med. Bull..

[125] Sasaki S., Shimokawa H. (1995). The amelogenin gene. Int. J. Dev. Biol..

[126] Li X., Liu D., Sun Y., Yang J., Yu Y. (2021). Association of genetic variants in enamel-formation genes with dental caries: A meta- and gene-cluster analysis. Saudi J. Biol. Sci..

[127] Sharmin N., Yuan J., Chow A.K. (2025). Using computer-generated protein models to analyze mutations linked to Amelogenesis Imperfecta. PLoS ONE.

[128] Chun Y.-H.P., Tan C., Villanueva O., Colley M.E., Quintanilla T.J., Basiouny M.S., Hartel C.A., Critchfield C.S., Bach S.B.H., Fajardo R.J. (2023). Overexpression of ameloblastin in secretory ameloblasts results in demarcated, hypomineralized opacities in enamel. Front. Physiol..

[129] Zhang Z., Zou X., Feng L., Huang Y., Chen F., Sun K., Song Y., Lv P., Gao X., Dong Y. (2023). Splicing mutations in AMELX and ENAM cause amelogenesis imperfecta. BMC Oral Health.

[130] Jedeon K., De la Dure-Molla M., Brookes S.J., Loiodice S., Marciano C., Kirkham J., Canivenc-Lavier M.-C., Boudalia S., Bergès R., Harada H. (2013). Enamel defects reflect perinatal exposure to bisphenol A. Am. J. Pathol..

[131] Bomfim G.H.S., Dupont G., Wright T., Mighell A., Lacruz R.S. (2025). Burden of hereditary enamel disorders. Trends Mol. Med..

[132] Thylstrup A., Fejerskov O. (1978). Clinical appearance of dental fluorosis in permanent teeth in relation to histologic changes. Community Dent. Oral Epidemiol..

[133] Limeback H. (1994). Enamel formation and the effects of fluoride. Community Dent. Oral Epidemiol..

[134] Robinson C., Connell S., Kirkham J., Brookes S.J., Shore R.C., Smith A.M. (2004). The effect of fluoride on the developing tooth. Caries Res..

[135] DenBesten P., Li W. (2011). Chronic fluoride toxicity: Dental fluorosis. Monogr. Oral Sci..

[136] Everett E.T. (2011). Fluoride’s effects on the formation of teeth and bones, and the influence of genetics. J. Dent. Res..

[137] Yamashita S., Okamoto M., Mendonca M., Fujiwara N., Kitamura E., Chang C.-S.S., Brueckner S., Shindo S., Kuriki N., Cooley M.A. (2024). Fluoride Alters Gene Expression via Histone H3K27 Acetylation in Ameloblast-like LS8 Cells. Int. J. Mol. Sci..

[138] Li J., Wang P., Gao J., Fei X., Liu Y., Ruan J. (2018). NaF Reduces KLK4 Gene Expression by Decreasing Foxo1 in LS8 Cells. Biol. Trace Elem. Res..

[139] Nagendra A.H., Bose B., Shenoy P.S. (2021). Recent advances in cellular effects of fluoride: An update on its signalling pathway and targeted therapeutic approaches. Mol. Biol. Rep..

[140] Samaranayake L., Porntaveetus T., Tsoi J., Tuygunov N. (2025). Facts and Fallacies of the Fluoride Controversy: A Contemporary Perspective. Int. Dent. J..

[141] Mascarenhas A.K. (2000). Risk factors for dental fluorosis: A review of the recent literature. Pediatr. Dent..

[142] Warren J.J., Levy S.M., Kanellis M.J. (2001). Prevalence of dental fluorosis in the primary dentition. J. Public Health Dent..

[143] Hubbard M.J., Mangum J.E., Perez V.A., Williams R. (2021). A Breakthrough in Understanding the Pathogenesis of Molar Hypomineralisation: The Mineralisation-Poisoning Model. Front. Physiol..

[144] Hubbard M.J., Perez V.A., Ganss B. (2021). 100 Years of Chalky Teeth Research: From Pioneering Histopathology to Social Good. Front. Dent. Med..

[145] Perez V.A., Mangum J.E., Hubbard M.J. (2020). Pathogenesis of Molar Hypomineralisation: Aged Albumin Demarcates Chalky Regions of Hypomineralised Enamel. Front. Physiol..

[146] Bekes K. (2020). Klinik, Diagnostik und Therapie der MIH. ZM Online.

[147] Almuallem Z., Busuttil-Naudi A. (2018). Molar incisor hypomineralisation (MIH)—An overview. Br. Dent. J..

[148] Childers N.K., Hubbard M.J. (2024). Adopting The D3 Group’s Translational Paradigm for Molar Hypomineralization and Chalky Teeth. Pediatr. Dent..

[149] Turner J.G. (1912). Two Cases of Hypoplasia of Enamel. Proc. R. Soc. Med..

[150] Slavkin H.C., Hu C.C., Sakakura Y., Diekwisch T., Chai Y., Mayo M., Bringas P., Simmer J., Mak G., Sasano Y. (1992). Gene expression, signal transduction and tissue-specific biomineralization during mammalian tooth development. Crit. Rev. Eukaryot. Gene Expr..

[151] Wright J.T., Carrion I.A., Morris C. (2015). The molecular basis of hereditary enamel defects in humans. J. Dent. Res..

[152] Paine M.L., White S.N., Luo W., Fong H., Sarikaya M., Snead M.L. (2001). Regulated gene expression dictates enamel structure and tooth function. Matrix Biol..

[153] Zhao H., Liu S., Wei Y., Yue Y., Gao M., Li Y., Zeng X., Deng X., Kotov N.A., Guo L. (2022). Multiscale engineered artificial tooth enamel. Science.

[154] Onuma K., Iijima M. (2017). Artificial enamel induced by phase transformation of amorphous nanoparticles. Sci. Rep..

[155] Zhang L., Zhang Y., Yu T., Peng L., Sun Q., Han B. (2022). Engineered Fabrication of Enamel-Mimetic Materials. Engineering.

[156] Lübke A., Enax J., Wey K., Fabritius H.-O., Raabe D., Epple M. (2016). Composites of fluoroapatite and methylmethacrylate-based polymers (PMMA) for biomimetic tooth replacement. Bioinspiration Biomim..

[157] Unterbrink P., Schulze zur Wiesche E., Meyer F., Fandrich P., Amaechi B.T., Enax J. (2024). Prevention of dental caries: A review on the improvements of toothpaste formulations from 1900 to 2023. Dent. J..

[158] Pawinska M., Paszynska E., Limeback H., Amaechi B.T., Fabritius H.-O., Ganss B., O’hAgan-Wong K., Wiesche E.S.Z., Meyer F., Enax J. (2024). Hydroxyapatite as an active ingredient in oral care: An international symposium report. Bioinspired Biomim. Nanobiomater..

[159] Amaechi B.T., AbdulAzees P.A., Alshareif D.O., Shehata M.A., Lima P.P.d.C.S., Abdollahi A., Kalkhorani P.S., Evans V. (2019). Comparative efficacy of a hydroxyapatite and a fluoride toothpaste for prevention and remineralization of dental caries in children. BDJ Open.

[160] Najibfard K., Ramalingam K., Chedjieu I., Amaechi B.T. (2011). Remineralization of early caries by a nano-hydroxyapatite dentifrice. J. Clin. Dent..

[161] Tschoppe P., Zandim D.L., Martus P., Kielbassa A.M. (2011). Enamel and dentine remineralization by nano-hydroxyapatite toothpastes. J. Dent..

[162] Amaechi B.T., Farah R., Liu J.A., Phillips T.S., Perozo B.I., Kataoka Y., Meyer F., Enax J. (2022). Remineralization of molar incisor hypomineralization (MIH) with a hydroxyapatite toothpaste: An in-situ study. BDJ Open.

[163] Pawinska M., Paszynska E., Amaechi B.T., Meyer F., Enax J., Limeback H. (2024). Clinical evidence of caries prevention by hydroxyapatite: An updated systematic review and meta-analysis. J. Dent..

[164] Reynolds E.C. (2009). Casein phosphopeptide-amorphous calcium phosphate: The scientific evidence. Adv. Dent. Res..

[165] Amaechi B.T., Yang K., Fandrich P., Wiesche E.S.Z., Enax J. Oral presentation: Efficacy of calcium hypophosphite in remineralizing initial caries in vitro. Proceedings of the 2025 IADR/PER General Session & Exhibition.

[166] Fandrich P., Hollmann B., Wiesche E.S.Z., Enax J. Poster presentation: Effects of calcium hypophosphite on human teeth in vitro: IADR/PER General Session & Exhibition. Proceedings of the 2025 IADR/PER General Session & Exhibition.

[167] Amaechi B.T., Vohra R., Abdollahi S., Yang K., Obiefuna A.C., Wiesche E.S.Z., Enax J. (2026). Remineralization of early caries lesions by calcium hypophosphite in vitro: A surface microhardness study. BDJ Open.

[168] Alkilzy M., Qadri G., Splieth C.H., Santamaría R.M. (2023). Biomimetic Enamel Regeneration Using Self-Assembling Peptide P11-4. Biomimetics.

[169] Gamea S., Radvar E., Athanasiadou D., Chan R.L., De Sero G., Ware E., Kundi S., Patel A., Horamee S., Hadadi S. (2025). Biomimetic Mineralization of Keratin Scaffolds for Enamel Regeneration. Adv. Healthc. Mater..

