# Supplementary material for: Human Enamel Formation: A Scoping Review for Oral Health Professionals
Source: Dent J (Basel). 2026 Jul 9;14(7):421. doi: 10.3390/dj14070421 (PMC13408100; doi:10.3390/dj14070421)
Supplement: Supplementary file 1 [file dentistry-14-00421-s001.zip › dentistry-4345975-supplementary.pdf]

Table S1: Results literature search.

#	Author	Title	Year	DOI (if available)
**1**	NYLEN, M. U.	ELECTRON MICROSCOPE AND ALLIED BIOPHYSICAL APPROACHES TO THE STUDY OF ENAMEL MINERALIZATION	1964	10.1111/j.1365-2818.1964.tb00522.x
**2**	Osborn, J. W.	Variations in structure and development of enamel	1973	
**3**	N.A.	Toxic effects of fluoride in enamel formation	1976	10.1111/j.1753-4887.1976.tb05681.x
**4**	Frank, R. M.	Electron microscope autoradiography of calcified tissues	1979	10.1016/s0074-7696(08)61823-9
**5**	Frank, R. M.	Tooth enamel: current state of the art	1979	10.1177/002203457905800220011
**6**	Thesleff, I.; Hurmerinta, K.	Tissue interactions in tooth development	1981	10.1111/j.1432-0436.1981.tb01107.x
**7**	Martin, L. B.; Boyde, A.; Grine, F. E.	Enamel structure in primates: a review of scanning electron microscope studies	1988	
**8**	Bawden, J. W.	Calcium transport during mineralization	1989	10.1002/ar.1092240212
**9**	Slavkin, H. C.	Positional signalling and patterning for amelogenesis in mouse molar tooth development	1989	10.3109/03008208909023878
**10**	Slavkin, H. C.	Molecular determinants during dental morphogenesis and cytodifferentiation: a review	1991	
**11**	Slavkin, H. C.; Hu, C. C.; Sakakura, Y.; Diekwisch, T.; Chai, Y.; Mayo, M.; Bringas, P.; Simmer, J.; Mak, G.; Sasano, Y.	Gene expression, signal transduction and tissue-specific biomineralization during mammalian tooth development	1992	
**12**	Kogaya, Y.	Sulfated glycoconjugates in amelogenesis. Comparative histochemistry and evolution of ectoderm-derived hard tissues	1994	
**13**	Limeback, H.	Enamel formation and the effects of fluoride	1994	10.1111/j.1600-0528.1994.tb01831.x

**14**	Brookes, S. J.; Robinson, C.; Kirkham, J.; Bonass, W. A.	Biochemistry and molecular biology of amelogenin proteins of developing dental enamel	1995	10.1016/0003-9969(94)00135-x
**15**	Deutsch, D.; Catalano-Sherman, J.; Dafni, L.; David, S.; Palmon, A.	Enamel matrix proteins and ameloblast biology	1995	10.3109/03008209509013710
**16**	Deutsch, D.; Palmon, A.; Dafni, L.; Catalano-Sherman, J.; Young, M. F.; Fisher, L. W.	The enamelin (tuftelin) gene	1995	
**17**	Goldberg, M.; Septier, D.; Lécolle, S.; Chardin, H.; Quintana, M. A.; Acevedo, A. C.; Gafni, G.; Dillouya, D.; Vermelin, L.; Thonemann, B.	Dental mineralization	1995	
**18**	Robinson, C.; Kirkham, J.; Brookes, S. J.; Bonass, W. A.; Shore, R. C.	The chemistry of enamel development	1995	
**19**	Simmer, J. P.; Fincham, A. G.	Molecular mechanisms of dental enamel formation	1995	10.1177/10454411950060020701
**20**	Takano, Y.	Enamel mineralization and the role of ameloblasts in calcium transport	1995	10.3109/03008209509016992
**21**	Wöltgens, J. H.; Lyaruu, D. M.; Bronckers, A. L.; Bervoets, T. J.; van Duin, M.	Biomineralization during early stages of the developing tooth in vitro with special reference to secretory stage of amelogenesis	1995	
**22**	Zeichner-David, M.; Diekwisch, T.; Fincham, A.; Lau, E.; Macdougall, M.; Moradian-Oldak, J.;	Control of ameloblast differentiation	1995	

	Simmer, J.; Snead, M.; Slavkin, H. C.			
**23**	Aoba, T.	Recent observations on enamel crystal formation during mammalian amelogenesis	1996	10.1002/(SICI)1097-0185(199606)245:2<208::AID-AR8>3.0.CO;2-S
**24**	Fincham, A. G.; Simmer, J. P.	Amelogenin proteins of developing dental enamel	1997	10.1002/9780470515303.ch9
**25**	Robinson, C.; Brookes, S. J.; Bonass, W. A.; Shore, R. C.; Kirkham, J.	Enamel maturation	1997	10.1002/9780470515303.ch11
**26**	Sasaki, T.; Takagi, M.; Yanagisawa, T.	Structure and function of secretory ameloblasts in enamel formation	1997	10.1002/9780470515303.ch4
**27**	Gibson, C. W.; Collier, P. M.; Yuan, Z. A.; Chen, E.	DNA sequences of amelogenin genes provide clues to regulation of expression	1998	10.1111/j.1600-0722.1998.tb02189.x
**28**	Kardos, T. B.	Enamel--an overview	1998	
**29**	Smith, C. E.	Cellular and chemical events during enamel maturation	1998	10.1177/10454411980090020101
**30**	Bartlett, J. D.; Simmer, J. P.	Proteinases in developing dental enamel	1999	10.1177/10454411990100040101
**31**	Fincham, A. G.; Moradian-Oldak, J.; Simmer, J. P.	The structural biology of the developing dental enamel matrix	1999	10.1006/jsbi.1999.4130
**32**	Gibson, C. W.	Regulation of amelogenin gene expression	1999	
**33**	Hubbard, M. J.	Calcium transport across the dental enamel epithelium	2000	10.1177/10454411000110040401
**34**	Moradian-Oldak, J.	Amelogenins: assembly, processing and control of crystal morphology	2001	10.1016/s0945-053x(01)00154-8
**35**	Paine, M. L.; White, S. N.; Luo, W.; Fong, H.; Sarikaya, M.; Snead, M. L.	Regulated gene expression dictates enamel structure and tooth function	2001	10.1016/s0945-053x(01)00153-6

**36**	Simmer, J. P.; Hu, J. C.	Dental enamel formation and its impact on clinical dentistry	2001	
**37**	Zeichner-David, M.	Is there more to enamel matrix proteins than biomineralization?	2001	10.1016/s0945-053x(01)00155-x
**38**	Goldberg, M.; Septier, D.	Phospholipids in amelogenesis and dentinogenesis	2002	10.1177/154411130201300305
**39**	Hubbard, Michael J.; Kon, Jew C.	Proteomic analysis of dental tissues	2002	10.1016/s1570-0232(02)00042-9
**40**	Moradian-Oldak, J.; Gharakhanian, N.; Jimenez, I.	Limited Proteolysis of Amelogenin: Toward Understanding the Proteolytic Processes in Enamel Extracellular Matrix	2002	10.1080/713713519
**41**	Papagerakis, P.; Berdal, A.; Mesbah, M.; Peuchmaur, M.; Malaval, L.; Nydegger, J.; Simmer, J.; Macdougall, M.	Investigation of osteocalcin, osteonectin, and dentin sialophosphoprotein in developing human teeth	2002	10.1016/s8756-3282(01)00683-4
**42**	Simmer, James P.; Hu, Jan C. C.	Expression, structure, and function of enamel proteinases	2002	10.1080/03008200290001159
**43**	Snead, Malcolm L.	Amelogenin protein exhibits a modular design: implications for form and function	2003	
**44**	Fukumoto, Satoshi; Yamada, Aya; Nonaka, Kazuaki; Yamada, Yoshihiko	Essential roles of ameloblastin in maintaining ameloblast differentiation and enamel formation	2005	10.1159/000091380
**45**	Paine, M. L.; Snead, M. L.	Tooth developmental biology: disruptions to enamel-matrix assembly and its impact on biomineralization	2005	10.1111/j.1601-6343.2005.00346.x
**46**	Sire, Jean-Yves; Delgado, Sidney; Fromentin, Delphine; Girondot, Marc	Amelogenin: lessons from evolution	2005	10.1016/j.archoralbio.2004.09.004

**47**	Bartlett, John D.; Ganss, Bernhard; Goldberg, Michel; Moradian-Oldak, Janet; Paine, Michael L.; Snead, Malcolm L.; Wen, Xin; White, Shane N.; Zhou, Yan L.	3. Protein-protein interactions of the developing enamel matrix	2006	10.1016/S0070-2153(06)74003-0
**48**	Margolis, H. C.; Beniash, E.; Fowler, C. E.	Role of macromolecular assembly of enamel matrix proteins in enamel formation	2006	10.1177/154405910608500902
**49**	Sire, Jean-Yves; Davit-Béal, Tiphaine; Delgado, Sidney; Gu, Xun	The origin and evolution of enamel mineralization genes	2007	10.1159/000102679
**50**	Lu, Yuhe; Papagerakis, Petros; Yamakoshi, Yasuo; Hu, Jan C-C; Bartlett, John D.; Simmer, James P.	Functions of KLK4 and MMP-20 in dental enamel formation	2008	10.1515/BC.2008.080
**51**	Gruenbaum-Cohen, Yael; Tucker, Abigail S.; Haze, Amir; Shilo, Dekel; Taylor, Angela L.; Shay, Boaz; Sharpe, Paul T.; Mitsiadis, Thimios A.; Ornoy, Asher; Blumenfeld, Anat; Deutsch, Dan	Amelogenin in cranio-facial development: the tooth as a model to study the role of amelogenin during embryogenesis	2009	10.1002/jez.b.21255
**52**	Feske, Stefan	CRAC channelopathies	2010	10.1007/s00424-009-0777-5
**53**	Lacruz, Rodrigo S.; Nanci, Antonio; Kurtz, Ira; Wright, J. Timothy; Paine, Michael L.	Regulation of pH During Amelogenesis	2010	10.1007/s00223-009-9326-7

**54**	Simmer, J. P.; Papagerakis, P.; Smith, C. E.; Fisher, D. C.; Rountrey, A. N.; Zheng, L.; Hu, J. C. C.	Regulation of dental enamel shape and hardness	2010	10.1177/0022034510375829
**55**	Moradian-Oldak, Janet	Protein-mediated enamel mineralization	2012	10.2741/4034
**56**	Sabel, N.	Enamel or primary teeth - morphological and chemical aspects	2012	
**57**	Simmer, James P.; Richardson, Amelia S.; Hu, Yuan-Yuan; Smith, Charles E.; Ching-Chun Hu, Jan	A post-classical theory of enamel biomineralization… and why we need one	2012	10.1038/ijos.2012.59
**58**	Bartlett, John D.	Dental enamel development: proteinases and their enamel matrix substrates	2013	10.1155/2013/684607
**59**	Lacruz, R. S.; Smith, C. E.; Kurtz, I.; Hubbard, M. J.; Paine, M. L.	New paradigms on the transport functions of maturation-stage ameloblasts	2013	10.1177/0022034512470954
**60**	Bartlett, John D.; Simmer, James P.	Kallikrein-related peptidase-4 (KLK4): role in enamel formation and revelations from ablated mice	2014	10.3389/fphys.2014.00240
**61**	Duan, X.	Ion channels, channelopathies, and tooth formation	2014	10.1177/0022034513507066
**62**	Ganss, Bernhard; Abbarin, Nastaran	Maturation and beyond: proteins in the developmental continuum from enamel epithelium to junctional epithelium	2014	10.3389/fphys.2014.00371
**63**	Jayasudha; Baswaraj; H K, Navin; K B, Prasanna	Enamel regeneration - current progress and challenges	2014	10.7860/JCDR/2014/10231.4883
**64**	Margolis, Henry C.; Kwak, Seo-Young; Yamazaki, Hajime	Role of mineralization inhibitors in the regulation of hard tissue biomineralization: relevance to initial enamel formation and maturation	2014	10.3389/fphys.2014.00339
**65**	Pugach, Megan K.; Gibson, Carolyn W.	Analysis of enamel development using murine model systems: approaches and limitations	2014	10.3389/fphys.2014.00313

**66**	Balic, Anamaria; Thesleff, Irma	Tissue Interactions Regulating Tooth Development and Renewal	2015	10.1016/bs.ctdb.2015.07.006
**67**	Bartlett, J. D.; Simmer, J. P.	New perspectives on amelotin and amelogenesis	2015	10.1177/0022034515572442
**68**	Fan, Yi; Zhou, Yachuan; Zhou, Xuedong; Xu, Xin; Pi, Caixia; Xu, Ruoshi; Zheng, Liwei	Epigenetic Control of Gene Function in Enamel Development	2015	10.2174/1574888x10666150305104730
**69**	Habelitz, S.	Materials engineering by ameloblasts	2015	10.1177/0022034515577963
**70**	Zhou, Yachuan; Zheng, Liwei; Sun, Jianxun; Ye, Ling; Zhou, Xuedong; Gao, Bo	Expression and Function of MicroRNAs in Enamel Development	2015	10.2174/1574888x10666150312101451
**71**	Sarkar, Juni; Wen, Xin; Simanian, Emil J.; Paine, Michael L.	V-type ATPase proton pump expression during enamel formation	2016	10.1016/j.matbio.2015.11.004
**72**	Jin, Ying; Wang, Chenglin; Cheng, Si; Zhao, Zhihe; Li, Juan	MicroRNA control of tooth formation and eruption	2017	10.1016/j.archoralbio.2016.08.026
**73**	Pham, Cong-Dat; Smith, Charles E.; Hu, Yuanyuan; Hu, Jan C-C; Simmer, James P.; Chun, Yong-Hee P.	Endocytosis and Enamel Formation	2017	10.3389/fphys.2017.00529
**74**	Yin, Kaifeng; Paine, Michael L.	Bicarbonate Transport During Enamel Maturation	2017	10.1007/s00223-017-0311-2
**75**	Kim, Hee-Eun; Hong, Jeong Hee	The overview of channels, transporters, and calcium signaling molecules during amelogenesis	2018	10.1016/j.archoralbio.2018.05.014
**76**	Varga, G.; DenBesten, P.; Rácz, R.; Zsembery, Á.	Importance of bicarbonate transport in pH control during amelogenesis - need for functional studies	2018	10.1111/odi.12738

**77**	Ali, Saqib; Farooq, Imran	A Review of the Role of Amelogenin Protein in Enamel Formation and Novel Experimental Techniques to Study its Function	2019	10.2174/0929866526666190731120018
**78**	Baranova, Juliana; Büchner, Dominik; Götz, Werner; Schulze, Margit; Tobiasch, Edda	Tooth Formation: Are the Hardest Tissues of Human Body Hard to Regenerate?	2020	10.3390/ijms21114031
**79**	Gil-Bona, Ana; Bidlack, Felicitas B.	Tooth Enamel and its Dynamic Protein Matrix	2020	10.3390/ijms21124458
**80**	Shaw, Wendy J.; Tarasevich, Barbara J.; Buchko, Garry W.; Arachchige, Rajith M. J.; Burton, Sarah D.	Controls of nature: Secondary, tertiary, and quaternary structure of the enamel protein amelogenin in solution and on hydroxyapatite	2020	10.1016/j.jsb.2020.107630
**81**	Costiniti, Veronica; Bomfim, Guilherme H.; Mitaishvili, Erna; Son, Ga-Yeon; Li, Yi; Lacruz, Rodrigo S.	Calcium Transport in Specialized Dental Epithelia and Its Modulation by Fluoride	2021	10.3389/fendo.2021.730913
**82**	Habelitz, S.; Bai, Y.	Mechanisms of Enamel Mineralization Guided by Amelogenin Nanoribbons	2021	10.1177/00220345211012925
**83**	Pandya, Mirali; Diekwisch, Thomas G. H.	Amelogenesis: Transformation of a protein-mineral matrix into tooth enamel	2021	10.1016/j.jsb.2021.107809
**84**	Shaik, Izaz; Dasari, Bhargavi; Shaik, Asma; Doos, Mina; Kolli, Hemanadh; Rana, Devyani; Tiwari, Rahul V. C.	Functional Role of Inorganic Trace Elements on Enamel and Dentin Formation: A Review	2021	10.4103/jpbs.jpbs_392_21

**85**	Simmer, James P.; Hu, Jan C-C; Hu, Yuanyuan; Zhang, Shelly; Liang, Tian; Wang, Shih-Kai; Kim, Jung-Wook; Yamakoshi, Yasuo; Chun, Yong-Hee; Bartlett, John D.; Smith, Charles E.	A genetic model for the secretory stage of dental enamel formation	2021	10.1016/j.jsb.2021.107805
**86**	Shahid, Shifa; Ikeda, Atsushi; Layana, Michelle C.; Bartlett, John D.	ADAM10: Possible functions in enamel development	2022	10.3389/fphys.2022.1032383
**87**	Wright, John Timothy	Enamel Phenotypes: Genetic and Environmental Determinants	2023	10.3390/genes14030545
**88**	Duverger, Olivier; Lee, Janice S.	The intricacies of tooth enamel: Embryonic origin, development and human genetics	2024	10.1016/j.jsb.2024.108135
**89**	Kegulian, Natalie C.; Visakan, Gayathri; Bapat, Rucha Arun; Moradian-Oldak, Janet	Ameloblastin and its multifunctionality in amelogenesis: A review	2024	10.1016/j.matbio.2024.05.007
**90**	Wu, Ke; Li, Xiaochan; Bai, Yunyang; Heng, Boon Chin; Zhang, Xuehui; Deng, Xuliang	The circadian clock in enamel development	2024	10.1038/s41368-024-00317-9
**91**	Hutami, Islamy R.; Arinawati, Dian Y.; Rahadian, Arief; Dewi, Rizqa C.; Rochmah, Yayun S.; Christiono, Sandy; Afroz, Shaista	Roles of calcium in ameloblasts during tooth development: A scoping review	2025	10.1016/j.jtumed.2024.12.010
**92**	Sasaki, S.; Shimokawa, H	The amelogenin gene		

Table S2: Preferred Reporting Items for Systematic reviews and Meta-Analyses extension for Scoping Reviews (PRISMA-ScR) Checklist

SECTION	ITEM	PRISMA-ScR CHECKLIST ITEM	REPORTED ON PAGE #
**TITLE**			
Title	1	Identify the report as a scoping review.	Title
**ABSTRACT**			
Structured summary	2	Provide a structured summary that includes (as applicable): background, objectives, eligibility criteria, sources of evidence, charting methods, results, and conclusions that relate to the review questions and objectives.	Abstract
**INTRODUCTION**			
Rationale	3	Describe the rationale for the review in the context of what is already known. Explain why the review questions/objectives lend themselves to a scoping review approach.	1.2
Objectives	4	Provide an explicit statement of the questions and objectives being addressed with reference to their key elements (e.g., population or participants, concepts, and context) or other relevant key elements used to conceptualize the review questions and/or objectives.	1.2
**METHODS**			
Protocol and registration	5	Indicate whether a review protocol exists; state if and where it can be accessed (e.g., a Web address); and if available, provide registration information, including the registration number.	NA
Eligibility criteria	6	Specify characteristics of the sources of evidence used as eligibility criteria (e.g., years considered, language, and publication status), and provide a rationale.	1.2
Information sources*	7	Describe all information sources in the search (e.g., databases with dates of coverage and contact with authors to identify additional sources), as well as the date the most recent search was executed.	1.2
Search	8	Present the full electronic search strategy for at least 1 database, including any limits used, such that it could be repeated.	1.2
Selection of sources of evidence†	9	State the process for selecting sources of evidence (i.e., screening and eligibility) included in the scoping review.	1.2
Data charting process‡	10	Describe the methods of charting data from the included sources of evidence (e.g., calibrated forms or forms that have been tested by the team before their use, and whether data charting was done independently or in duplicate) and any processes for obtaining and confirming data from investigators.	NA
Data items	11	List and define all variables for which data were sought and any assumptions and simplifications made.	NA
Critical appraisal of individual sources of evidence§	12	If done, provide a rationale for conducting a critical appraisal of included sources of evidence; describe the methods used and how this information was used in any data synthesis (if appropriate).	NA
Synthesis of results	13	Describe the methods of handling and summarizing the data that were charted.	NA

SECTION	ITEM	PRISMA-ScR CHECKLIST ITEM	REPORTED ON PAGE #
**RESULTS**			
Selection of sources of evidence	14	Give numbers of sources of evidence screened, assessed for eligibility, and included in the review, with reasons for exclusions at each stage, ideally using a flow diagram.	2.2
Characteristics of sources of evidence	15	For each source of evidence, present characteristics for which data were charted and provide the citations.	Sup. S1
Critical appraisal within sources of evidence	16	If done, present data on critical appraisal of included sources of evidence (see item 12).	NA
Results of individual sources of evidence	17	For each included source of evidence, present the relevant data that were charted that relate to the review questions and objectives.	NA
Synthesis of results	18	Summarize and/or present the charting results as they relate to the review questions and objectives.	3.-4.
**DISCUSSION**			
Summary of evidence	19	Summarize the main results (including an overview of concepts, themes, and types of evidence available), link to the review questions and objectives, and consider the relevance to key groups.	NA
Limitations	20	Discuss the limitations of the scoping review process.	7.
Conclusions	21	Provide a general interpretation of the results with respect to the review questions and objectives, as well as potential implications and/or next steps.	8.
**FUNDING**			
Funding	22	Describe sources of funding for the included sources of evidence, as well as sources of funding for the scoping review. Describe the role of the funders of the scoping review.	NA

JBI = Joanna Briggs Institute; PRISMA-ScR = Preferred Reporting Items for Systematic reviews and Meta-Analyses extension for Scoping Reviews.
* Where *sources of evidence* (see second footnote) are compiled from, such as bibliographic databases, social media platforms, and Web sites.
† A more inclusive/heterogeneous term used to account for the different types of evidence or data sources (e.g., quantitative and/or qualitative research, expert opinion, and policy documents) that may be eligible in a scoping review as opposed to only studies. This is not to be confused with *information sources* (see first footnote).
‡ The frameworks by Arksey and O'Malley (6) and Levac and colleagues (7) and the JBI guidance (4, 5) refer to the process of data extraction in a scoping review as data charting.
§ The process of systematically examining research evidence to assess its validity, results, and relevance before using it to inform a decision. This term is used for items 12 and 19 instead of "risk of bias" (which is more applicable to systematic reviews of interventions) to include and acknowledge the various sources of evidence that may be used in a scoping review (e.g., quantitative and/or qualitative research, expert opinion, and policy document).

From: Tricco AC, Lillie E, Zarin W, O'Brien KK, Colquhoun H, Levac D, et al. PRISMA Extension for Scoping Reviews (PRISMAScR): Checklist and Explanation. Ann Intern Med. 2018;169:467–473. doi: 10.7326/M18-0850.
